# A JAK Inhibitor for Treatment of Rheumatoid Arthritis: The Baricitinib Experience

**DOI:** 10.3390/jcm12134527

**Published:** 2023-07-06

**Authors:** Peter C. Taylor, Cedric Laedermann, Rieke Alten, Eugen Feist, Ernest Choy, Ewa Haladyj, Inmaculada De La Torre, Pascal Richette, Axel Finckh, Yoshiya Tanaka

**Affiliations:** 1 Botnar Research Centre, Nuffield Department of Orthopaedics, Rheumatology, and Musculoskeletal Sciences, University of Oxford, Oxford OX3 7LD, UK; 2 Eli Lilly and Company, Indianapolis, IN 46285, USA; laedermann_cedric@lilly.com (C.L.); haladyj_ewa@lilly.com (E.H.); ide_la_torre@lilly.com (I.D.L.T.); 3 Internal Medicine II, Rheumatology, SCHLOSSPARK-KLINIK, University Medicine Berlin, 14059 Berlin, Germany; rieke.alten@parkkliniken.de; 4 Department of Rheumatology, Helios Clinic Vogelsang-Gommern, Cooperation Partner of the Otto-von-Guericke University Magdeburg, 39245 Magdeburg, Germany; eugen.feist@charite.de; 5 Division of Infection and Immunity, Cardiff University School of Medicine, Cardiff CF14 4YS, UK; choyeh@cardiff.ac.uk; 6 Service de Rhumatologie, Hôpital Lariboisière, 75010 Paris, France; pascal.richette@aphp.fr; 7 Inserm, UMR-S 1132, Bioscar, Université de Paris, 75010 Paris, France; 8 Division of Rheumatology, Department of Medicine, Geneva University Hospitals, 1205 Geneva, Switzerland; axel.finckh@hcuge.ch; 9 First Department of Internal Medicine, University of Occupational and Environmental Health, Kitakyushu 807-0804, Japan; tanaka@med.uoeh-u.ac.jp

**Keywords:** baricitinib, rheumatoid arthritis, real-world evidence, randomised controlled trial

## Abstract

Baricitinib, an oral selective Janus kinase (JAK)1/JAK2 inhibitor, is approved as monotherapy or in combination with methotrexate for treating adults with moderate-to-severe active rheumatoid arthritis (RA) and provides improvements in clinical signs, symptoms and patient-reported outcomes. Currently, baricitinib is approved for treating RA in more than 75 countries. In several pivotal Phase II and III RA trials (RA-BALANCE, RA-BEGIN, RA-BEAM, RA-BUILD, RA-BEACON, RA-BEYOND), up to seven years of baricitinib treatment was well tolerated and provided rapid and sustained efficacy, which was confirmed in real-world settings. Safety signals for another JAK inhibitor, tofacitinib, have emerged, as observed in the post-marketing Phase IIIb/IV trial Oral Rheumatoid Arthritis Trial (ORAL) Surveillance; safety signals were subsequently highlighted in a retrospective study of baricitinib and consequently new recommendations and warnings and precautions for all JAK inhibitors have been issued. Ongoing studies to further characterise and clarify the benefit:risk of JAK inhibitors include registries and controlled trials. This capstone review summarises clinical and real-world data outlining the benefit:risk profile of baricitinib, confirming that the improved disease activity and physical function of patients with RA treated with this JAK inhibitor observed in clinical trials is translated into effectiveness in clinical practice, with a low rate of discontinuations.

## 1. Introduction

After two decades during which biologic disease-modifying antirheumatic drugs (bDMARDs) revolutionised the treatment and prognosis of patients with rheumatoid arthritis (RA), a new class of targeted synthetic DMARDS (tsDMARDs), Janus kinase inhibitors (JAKis), have been recognised by international societies as effective and well-tolerated treatment options. While conventional synthetic DMARDs (csDMARDs) remain the recommended first-line ‘anchor therapy’ for patients with early RA [1,2], updated European Alliance of Associations for Rheumatology (EULAR) management guidelines recommend adding a bDMARD or a JAKi to csDMARD therapy if symptoms are not sufficiently improved. JAKis should be considered only after risk assessment (see also Section 3.3. Safety below) but, in common with interleukin (IL)-6 inhibitors, may be favoured if combination therapy is contraindicated [2].

JAKis are small-molecule, targeted oral treatments with a rapid onset of action [3]. Baricitinib, an oral selective, and reversible, JAK1/JAK2 inhibitor was the first in the JAKi class to be approved to treat RA in the European Union (EU) [4] based on the demonstration of superior efficacy compared with placebo and tumour necrosis factor inhibitors (TNFis) in populations with an inadequate response (IR) to csDMARDs, including methotrexate (MTX) [5,6]. Baricitinib also demonstrated superior efficacy to MTX monotherapy in patients who had received no or minimal csDMARDs and who were naïve to bDMARDs [7] and was associated with clinical improvements in patients with an IR to bDMARDs [8].

Baricitinib’s first global approval was in Japan, then the EU in 2017, as monotherapy or in combination with MTX for the treatment of adults with moderate-to-severe active RA who have responded inadequately to, or who are intolerant of one or more DMARDs [4,9]. Following this, approval was received in the United States (US) in 2018 for the treatment of adults with moderately to severely active RA who have had an IR to one or more TNFis [10]. Baricitinib is now licenced in more than 75 countries for the treatment of RA. In some markets, baricitinib is also approved as therapy for atopic dermatitis, COVID-19 and/or alopecia areata [9,10].

Five years after entering the market, evidence for baricitinib in long-term (up to seven years) extension studies of integrated data sets and real-world data are available and new data are emerging. Within this review, both clinical trial data and all current real-world data for baricitinib in the treatment of RA published to date are summarised and discussed.

### 1.1. Pharmacodynamic and Pharmacokinetic Properties of Baricitinib

In vitro, baricitinib inhibits JAK1 (half-maximal inhibitory concentration [IC_50_] 5.9 nmol/L) and JAK2 (IC_50_ 5.7 nmol/L) but has lower potency against tyrosine kinase 2 (IC_50_ 53 nmol/L) and JAK3 (IC_50_ 560 nmol/L) [4]. In vitro analyses also showed that baricitinib inhibited JAK1/3 signalling to a lesser extent than upadacitinib and tofacitinib, with all three JAKis inhibiting the signalling of JAK2/2-dependent cytokines [11]. In the Phase III trial, RA-BUILD [5], patients demonstrated reductions in levels of biomarkers associated with synovial inflammation (C1M, C3M and C4M) and bone resorption (CTX-I), which together with clinical improvements and radiographic data suggests that baricitinib inhibits key pathological processes in RA [12]. A study assessing the effects of baricitinib and tofacitinib in mice also revealed that JAK inhibition increases bone mass, while the robust up-regulation of markers for osteoblast function—such as osteocalcin and Wnt signalling—were seen in osteoblasts exposed to JAKis in vitro; the anabolic effect of JAKis was illustrated by the stabilisation of β-catenin [13]. In people with RA, JAK inhibition induced bone-anabolic effects as evidenced by the repair of arthritic bone erosions after treatment with tofacitinib [13]. More recently, a prospective study reported for the first time that baricitinib treatment increases resistance to mechanical load, with estimated failure load and bone stiffness correlated with reduced cortical porosity, thereby improving the functional properties of bone [14].

The relatively short half-life of baricitinib (12.5 h [9]), and convenient once daily oral administration, brought a substantial change to the current treatment options for RA. Improvements in the American College of Rheumatology 20% (ACR20) response rate, the Disease Activity Score in 28 joints (DAS28) and other clinical outcomes were seen as early as the first week with baricitinib treatment as compared to MTX [7,9]. In humans, approximately 75% of the administered baricitinib dose is cleared in the urine and about 20% in the faeces; drug metabolism is mediated by CYP3A4, with <10% of the dose identified as undergoing biotransformation [9] (Table 1 [10,15,16,17,18,19,20,21,22,23,24,25,26]). This has a direct clinical implication in patients with concomitant mild-to-moderate or moderate-to-severe renal impairment, who should receive a 2 mg dose in some circumstances [9]. As a result of moderate hepatic metabolism, baricitinib has limited potential for drug–drug interactions, with interactions evident only with probenecid (a strong organic anion transporter-3 [OAT3] inhibitor) [27]. This is in contrast to most other JAKis, which are associated with numerous drug–drug interactions (Table 1). Thus, in patients concomitantly receiving baricitinib and medications metabolised by the liver—such as ketoconazole, fluconazole or rifampicin—no dose adjustment is required. The synthetic nature of baricitinib eliminates the risk of immunogenicity (neutralising antibody response) and the temporary interruption of baricitinib does not cause a loss of response [28]. Importantly, when there is a need for drug discontinuation, 90% of the baricitinib dose is eliminated within 24 h [9]. Although baricitinib is contraindicated during pregnancy, in contrast to many other available therapies, it may be stopped as late as one week prior to a planned pregnancy [9].

### 1.2. Dose Flexibility

Baricitinib is available in two dosage strengths (2 mg and 4 mg), each administered once daily. Baricitinib 4 mg is the indicated starting dose, with potential to taper to 2 mg, in all countries except the US, Canada and China, where a dose of 2 mg is approved for treating RA. Patients aged ≥65 years and those with a history of chronic or recurrent infections should receive a dose of 2 mg according to EU recommendations [9]. Both baricitinib 4 mg and 2 mg are efficacious among different treatment populations [5,8,9,29].

Elderly patients often have comorbidities and require multiple concomitant medications, with concerns that treatments will be less effective, and the number of adverse events (AEs) increased [30]. Subgroup analysis of data from two Phase III trials in patients with RA and an IR to csDMARDs (csDMARD-IR)—RA-BUILD [5] and RA-BEAM [6]—demonstrated similar clinical efficacy with baricitinib 4 mg once daily in patients aged <50 years and those aged ≥65 years [30]. In general, while efficacy is not affected by age, safety might be diminished, with higher rates of discontinuation due to AEs in elderly patients aged ≥65 years, mainly related to an increased risk of serious infections [30]. A reduced dosage of baricitinib 2 mg/day is recommended in patients aged ≥65 years and those with moderate renal impairment, and may be appropriate for patients with a history of chronic or recurrent infections [9], and is also in line with the European Medicines Agency (EMA) Pharmacovigilance Risk Assessment Committee (PRAC) recommendation to use a lower effective dose for all JAKs across all indications when treating populations at risk of venous thromboembolism (VTE), cancer or major cardiovascular (CV) problems [31]. Therefore, based on extrapolation from the Oral Rheumatoid Arthritis Trial (ORAL) Surveillance [32] and the results of a retrospective analysis of baricitinib data [33], the lower dose of baricitinib 2 mg is recommended for all patients at risk of AEs of special interest (CV events, malignancy and deep vein thrombosis), with the possibility to step up to a higher baricitinib dose of 4 mg if disease activity is not controlled.

## 2. Efficacy across RA Populations in Randomised Controlled Trials (RCTs)

### 2.1. RA Populations

In Phase III RCTs conducted in countries across the world, baricitinib was evaluated in a range of patients with RA, including those who were bDMARD-naïve in RA-BEGIN [7], MTX-IR in RA-BEAM [6] and RA-BALANCE [34,35], csDMARD-IR in RA-BUILD [5] and bDMARD-IR (27% ≥3 prior bDMARDs) in RA-BEACON [8]. Baricitinib has been evaluated as monotherapy or in combination with MTX, with or without concomitant glucocorticoids.

### 2.2. Clinical Outcomes

In RA-BEGIN, baricitinib 4 mg/day, as monotherapy or in combination with MTX, had a rapid response with superior and improved efficacy outcomes (ACR20 at 24 weeks), clinically and functionally, when directly compared with MTX monotherapy in patients predominantly naïve to csDMARDs. Rates of remission (REM) and low disease activity (LDA) were also higher with both baricitinib regimens than with MTX [7].

In RA-BALANCE [34,35], RA-BUILD [5] and RA-BEAM [6], baricitinib was established as a treatment option for patients with an IR to one or more csDMARDs. In each trial, baricitinib showed significant rapid improvements in ACR20 response rates and other clinical outcomes relative to placebo [5,6,35], and for the first time, in RA-BEAM, versus adalimumab [6]. Specifically, in patients on background MTX, baricitinib 4 mg/day was the first JAKi to demonstrate clinical superiority by 12 weeks versus adalimumab with regard to the proportion of patients achieving ACR20 and DAS28-C-reactive protein (DAS28-CRP) change from baseline. In addition, patients treated with baricitinib demonstrated higher rates of REM (Simplified Disease Activity Index [SDAI] ≤3.3) as compared to adalimumab at 12 weeks [6].

In RA-BEACON, patients with an IR or intolerance to bDMARDs had significant functional and clinical improvements with baricitinib treatment, achieving greater benefits than those receiving placebo [8]. REM at 12 weeks, assessed by improvements in DAS28-CRP, was achieved at a significantly higher rate with baricitinib 2 mg/day and 4 mg/day than with placebo [8]. A post hoc analysis [36] reported that baricitinib was effective and superior to placebo, regardless of the previous number or type of concomitant bDMARDs, or glucocorticoid use.

**Figure 1:**
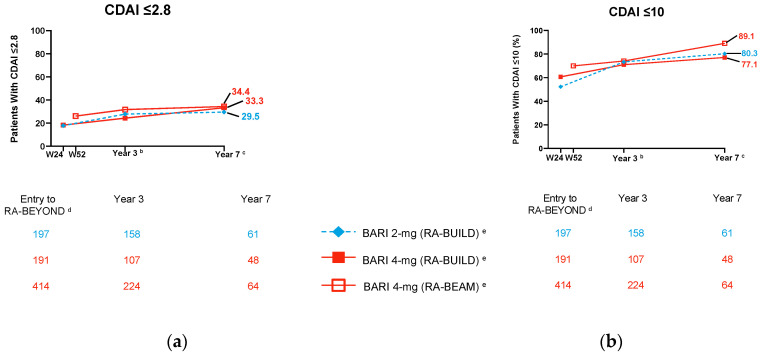
Long-term efficacy of baricitinib in RA-BEYOND according to originating study (RA-BEAM or RA-BUILD) ^a^: (**a**) CDAI remission (≥2.8); (**b**) CDAI LDA (≥10). Adapted from a presentation by Caporali and colleagues [37]. ^a^ Data were also collected in RA-BEYOND from patients randomised to placebo or adalimumab in the originating studies; the data shown here are from patients in the baricitinib treatment groups for the entire study duration. ^b^ Year three corresponds to 156 weeks of the originating study RA-BUILD and 160 weeks of the originating study RA-BEAM. ^c^ Year seven corresponds to 360 weeks of the originating study RA-BUILD and 364 weeks of the originating study RA-BEAM. ^d^ Entry to RA-BEYOND was at 24 weeks of the originating study RA-BUILD and 52 weeks of the originating study RA-BEAM. ^e^ Patients were analysed by treatment assigned at randomisation in the originating study, regardless of rescue. CDAI, Clinical Disease Activity Index; LDA, low disease activity; W, week.

In the long-term extension trial RA-BEYOND, baricitinib maintained efficacy and normative physical function for up to almost seven years in all three populations investigated (csDMARD-IR, including MTX-IR, and bDMARD-IR patients) (Figure 1) [37,38].

There are currently no head-to-head trials comparing the efficacy of the JAKis. However, in non-inferiority studies, only baricitinib 4 mg [6] and upadacitinib 15 mg [39] have shown superiority to adalimumab in prespecified endpoints, whereas tofacitinib [40] and filgotinib have not [41].

### 2.3. Radiographic Outcomes

Before the era of bDMARDs, radiographic progression was the main reason for disability and early retirement in difficult-to-treat (D2T) patients with RA. With new treatment options and a treat-to-target approach focused on the reduction of inflammation, rapid achievement of REM or LDA is accompanied by no or limited structural joint damage [1,2].

In csDMARD-naïve patients (RA-BEGIN), radiographic progression seen with baricitinib plus MTX was significantly less than that seen with MTX monotherapy both at 24 and 52 weeks [7]. Post hoc analysis showed that patients achieving sustained clinical response (DAS28-high-sensitivity CRP ≤3.2 or SDAI score ≤11) at 52 weeks were less likely to experience structural damage progression and that patients achieving these responses were less likely to progress if in the baricitinib or the baricitinib plus MTX group compared to the MTX monotherapy group [42]. In MTX-IR patients (RA-BEAM), the inhibition of radiographic progression (modified total Sharp score [mTSS]) was significantly greater with baricitinib than with placebo at 24 weeks [6]. Progression of bone erosion and joint space narrowing were each similarly inhibited by baricitinib and adalimumab. Interestingly, in a post hoc analysis of RA-BEGIN and RA-BEAM, reduced structural progression (mTSS) was observed regardless of disease activity control (Clinical Disease Activity Index [CDAI] REM and LDA versus CDAI moderate and high disease activity) in baricitinib-treated patients, but not in those who received placebo or MTX [43], providing evidence of an uncoupling of the link between disease activity and radiographic progression previously demonstrated with TNFis, IL-6 inhibitors and rituximab [44,45,46].

In the seven-year, long-term extension study, RA-BEYOND [47], which included patients initially randomised in RA-BEGIN [7], RA-BEAM [6] or RA-BUILD [5], approximately 40–72% of patients, depending on their originating study and initial treatment, treated with baricitinib 2 mg or 4 mg/day (monotherapy or combined with a csDMARD) had no radiographic progression (threshold of mTSS ≤ 0) over five years [47].

### 2.4. Patient-Reported Outcomes: Pain and Physical Function

Typically, clinicians focus on composite disease activity scores to assess disease control with a view to preventing structural progression and optimising function in RA. However, for many patients with RA, pain and fatigue are dominant symptoms [48,49]. Pain and impairment of other subjective quality of life measures may persist even after clinical targets for disease activity are achieved [50,51,52].

Patients treated with baricitinib (monotherapy or in combination with MTX) demonstrated more rapid and greater pain relief relative to placebo, MTX monotherapy and adalimumab in RCTs [5,6,36,53]. Relative to MTX monotherapy, baricitinib provided improvements in pain, health-related quality of life, Patient’s Global Assessment of Disease Activity, fatigue and work and activity impairments in RA-BEGIN [54]. Additionally, patients treated with baricitinib in RA-BEAM demonstrated greater improvements in physical function than adalimumab, with more patients achieving Health Assessment Questionnaire–Disability Index (HAQ-DI) score improvement ≥0.22 or ≥0.3 at 24 weeks, an improvement considered as the minimally clinically important difference [6]. Furthermore, baricitinib-treated patients in RA-BEAM experienced a significant reduction in pain as compared to those treated with adalimumab as early as 2 weeks and tiredness as early as 8 weeks [6], which was associated with improved daily activity and work productivity by 12 and 24 weeks [55]. Post hoc analysis of RA-BEAM demonstrated that the median time needed for an improvement in pain of ≥50% was 4 weeks for baricitinib and 7.9 weeks with adalimumab [56]. In patients with uncontrolled inflammation after 24 weeks, as measured by a CRP level >10 mg/L, mean improvement in pain with baricitinib was significantly larger than with either adalimumab or placebo, with the study authors suggesting an effect of baricitinib on pain irrespective of inflammation control [56]. In exploratory analyses of these data, patients in LDA or REM (DAS28-erythrocyte sedimentation rate [DAS28-ESR]) had improvements in pain and HAQ-DI scores with baricitinib that were significantly larger than those seen with both placebo and adalimumab [57]. This finding led the authors to suggest that, in patients with well-controlled RA, baricitinib may exhibit effects beyond immunomodulation [57]. In a matching-adjusted indirect comparative analysis, the efficacy of several DMARDs on pain and HAQ-DI was assessed [54]. With the three matching methods used, baricitinib monotherapy consistently showed greater reductions in pain and improved physical function than tocilizumab and adalimumab monotherapy [54]. Statistically significant differences between baricitinib and tofacitinib treatment in pain reduction and improved physical function were not consistently observed [54]. Significantly greater improvements in most patient-reported outcomes (Short Form-36 Physical Component Summary, EQ-5D 5L components, Functional Assessment of Chronic Illness Therapy-Fatigue, Patient Global Assessment [PGA] and visual analogue scale [VAS] scores for pain) were experienced at 24 weeks with baricitinib 2 mg/day and 4 mg/day versus placebo in bDMARD-IR patients (RA-BEACON [58]).

As structural damage, reduced functionality and associated pain and reduction in quality of life can begin early in the disease course, the superiority of baricitinib to TNFi therapy may be of interest to healthcare providers and patients.

### 2.5. Temporary Treatment Interruption and Tapering

Temporary interruption to RA therapy is a common occurrence in clinical trials due to factors such as non-compliance, the necessity for surgery or AEs [28]. Analysis of the Phase III baricitinib studies RA-BEAM, RA-BUILD and RA-BEACON showed that 8.5–18.1% of patients interrupted their treatment through 24 weeks and that the most common cause of baricitinib treatment interruption was AEs (79.2–91.9% of interruptions), with treatment interruption lasting less than 2 weeks and generally occurring within the first two months of baricitinib treatment. In MTX-naïve patients, the first interruption occurred on average at 4–5 months [28]. During the brief baricitinib interruptions, RA symptoms increased in many patients (>59% for morning joint stiffness [MJS] duration and 86–90% for MJS severity, worst tiredness and worst joint pain), albeit minimally (increase of ≤30 min for MJS duration, increase of ≤2 units for other outcomes), and after the resumption of baricitinib treatment, symptoms resolved. Although AEs were the most frequent reason for baricitinib treatment interruption, these events infrequently reoccurred once treatment was restarted [28]. This indicates that AEs were usually short-term and are unlikely to impede efficacy, consistent with long-term efficacy outcomes that were not impacted by treatment interruptions [28].

Guidelines from the American College of Rheumatology (ACR) [1] and the EULAR [2] recommend that tapering DMARD therapy may be considered in patients who achieve sustained REM. Results from the long-term study RA-BEYOND demonstrated that patients with sustained LDA or REM on baricitinib 4 mg/day for ≥15 months, who then continued on baricitinib 4 mg/day or tapered to 2 mg/day, maintained LDA (80% 4  mg; 67% 2 mg) and REM (40% 4 mg; 33% 2 mg) at 48 weeks [59]. Dose reduction resulted in small, yet statistically significant increases in disease activity at 12, 24 and 48 weeks and produced earlier and more frequent relapse (loss of step-down criteria) over 48 weeks compared with 4 mg maintenance (23% 4 mg vs. 37% 2 mg, *p* = 0.001). At the same time, patients tapered to baricitinib 2 mg/day had numerically fewer infections, including non-serious infections, than those who continued on baricitinib 4 mg/day [59]. Overall, RA-BEYOND showed that baricitinib 2 mg/day provided acceptable efficacy, with only one in five patients returned to the 4 mg/day dose, which then resulted in disease control similar to that prior to tapering in the vast majority [59]. This may be of particular interest for managing the treatment of patients with safety concerns.

## 3. Baricitinib in Real-World Settings

### 3.1. Characteristics of Baricitinib-Treated Patients in Real-World Settings

Real-world evidence (RWE) helps health authorities and healthcare providers (HCPs) gain further insights regarding treatment effectiveness and safety in real-life settings as they focus on heterogenous patient populations (who can be older, more treatment refractory, with more comorbid conditions and with more co-treatments than clinical trial populations). RWE permits the identification of associations between treatment and rare AEs and informs whether a drug’s performance as assessed in RCTs is recapitulated in real-life settings. It also helps the medical community to better understand treatment patterns, such as line of therapy utilisation, or to define risks associated with lower effectiveness or poorer drug survival. Furthermore, RWE helps HCPs understand whether drug utilisation reflects official guidelines such as those of EULAR [2] and ACR [1]. Finally, RWE allows for longer drug exposure observations and comparisons with greater numbers of other drugs than RCTs typically provide. Below is a comprehensive descriptive review of the literature regarding RWE for baricitinib that has been reported to date; data were sourced from registries, monocentric and multicentric clinical practice studies, administrative or insurance claim databases, or prospective observational studies. At the time of writing, 41 published sources for RWE of baricitinib in the treatment of RA were identified [29,33,60,61,62,63,64,65,66,67,68,69,70,71,72,73,74,75,76,77,78,79,80,81,82,83,84,85,86,87,88,89,90,91,92,93,94,95,96,97,98].

Due to the heterogeneity between each data source—such as patient characteristics and outcomes reported—a meta-analysis was not feasible.

Supplementary Table S1 summarises available baseline characteristics of baricitinib-treated patients in real-world settings [29,33,60,61,62,63,64,65,66,67,68,69,70,71,72,73,74,75,76,77,78,79,80,81,82,83,84,85,86,87,88,89,90,91,92,93,94,95,96,97,98]. The typical patient in the various RWE sources is aged about 60 years (which is older than the mean age of patients in baricitinib RCTs). In the RWE sources, only a limited number of patients were bio-naïve and most tended to have several b/tsDMARD failures before initiating baricitinib. In an observational Italian prospective study (IPS), patients treated with baricitinib who were bDMARD-naïve were younger with shorter disease duration and presented with fewer comorbidities, including CV disease, hypercholesterolaemia and diabetes, than those who were bDMARD-IR [64]. A study from the British Society for Rheumatology Biologics Register for Rheumatoid Arthritis (BSRBR-RA) also showed that bDMARD-naïve patients treated with baricitinib had shorter disease duration and numerically fewer comorbidities compared with the bDMARD-IR patients [29]. The proportion of patients treated with baricitinib monotherapy varies significantly between countries; for example, the proportion was 43% in Spain (ORBIT-RA registry) [60] and 87% in Denmark (DANBIO registry) [66] but is overall around 50%. With regard to dosage, most patients were using 4 mg (see Supplementary Table S1) and one study in bio-naïve patients included in a Spanish registry (BIO-1) reported that patients who received baricitinib 2 mg were older than those who received baricitinib 4 mg (73 years vs. 56 years, *p* < 0.0001) [61]. An older age of patients treated with baricitinib 2 mg versus 4 mg (67.9 years vs. 61.7 years) was also identified in a Japanese post-marketing surveillance (PMS) study in which >70% of patients had an IR to at least one prior b/tsDMARD [76], as well as in the BSRBR-RA (67.6 years vs. 58.7 years) [29]. This reflects that rheumatologists are cautious and apply label recommendations for a lower baricitinib dosage when treating elderly patients.

### 3.2. Treatment Outcomes for Baricitinib in Real-Life Settings

**Figure 2:**
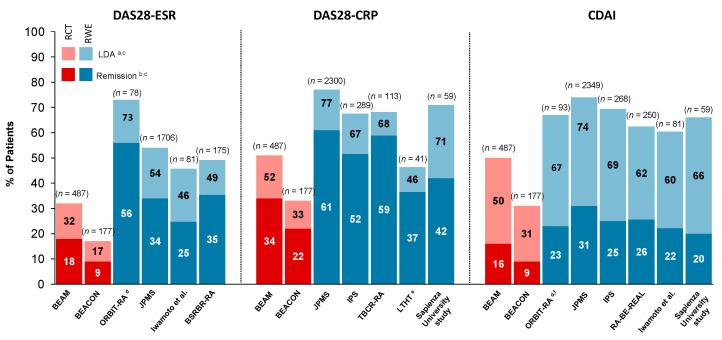
Treat-to-target goals of remission/LDA with BARI at six months of treatment in real-world studies and in RCTs in patients with RA. The included studies are: BEAM [6], BEACON [8], BSRBR-RA [29], ORBIT-RA [60], IPS [64], TBCR-RA [74], Iwamoto et al. [75], JPMS [76], RA-BE-REAL [77], Sapienza University study [78], LTHT [81]. ^a^ LDA was defined as DAS28-CRP ≤3.2, DAS28-ESR ≤3.2, CDAI ≤10. ^b^ Remission was defined as DAS28-CRP <2.6, DAS28-ESR <2.6, CDAI ≤2.8. ^c^ Blue bars indicate RWE reporting a pooled BARI dose group and red bars indicate RCT data from patients on BARI 4 mg. ^d^ REM value extrapolated from a graph [60]. ^e^ LDA was defined as DAS28-CRP ≤3.1. ^f^ LDA was defined as CDAI ≤11.0; remission was defined as CDAI ≤3.3. BARI, baricitinib; BSRBR-RA, British Society for Rheumatology Biologics Register for Rheumatoid Arthritis; CDAI, Clinical Disease Activity Index; DAS28-CRP, Disease Activity Score in 28 joints—C-reactive protein; DAS28-ESR, Disease Activity Score in 28 joints—erythrocyte sedimentation rate; IPS, Italian prospective study; JPMS, Japan post-marketing surveillance; LDA, low disease activity; LTHT, Leeds Teaching Hospitals NHS Trust database; *n* = number of patients in the study-specific baricitinib group; RA, rheumatoid arthritis; RCT, randomised controlled trial; RWE, real-world evidence; TBCR, Tsurumai Biologics Communication Registry.

Despite baricitinib use in patients predominantly refractory to previous cs/b/tsDMARD treatment, baricitinib demonstrated effectiveness in the majority of patients, as reflected by DAS28-ESR, DAS28-CRP and CDAI LDA and REM rates after six months of treatment (Figure 2). Significant improvements were reported in two Italian monocentric studies, which used clinical and ultrasound evaluation (at Careggi University Hospital in Florence and at Sapienza University of Rome in Rome) as early as at one month for joint scores and other clinometric scores [78,83], as well as in a Japanese registry, Tsurumai Biologics Communication Registry of RA patients (TBCR-RA), for DAS28-CRP change from baseline [74]. In one of the monocentric studies from Italy, the clinical response to baricitinib was not affected by previous bDMARD exposure [78]. Five observational studies—two prospective multicentric studies from Italy, the IPS [64] and an analysis at Sapienza University of Rome [78]; a prospective, monocentric real-world analysis in Erlangen, Germany [82]; a Lilly-conducted multicentre prospective study [77]; and a study based on data from a Spanish registry, ORBIT-RA [60]—all confirmed that baricitinib alleviates pain, while the Lilly-conducted study and a study using data from the Swedish Rheumatology Quality (SRQ) register by the Anti-Rheumatic Therapy in Sweden (ARTIS) Study Group reported that baricitinib improves physical function, as highlighted by improvements in HAQ-DI scores [65,77].

#### 3.2.1. Factors Affecting Treatment Outcomes—Drug Discontinuation, Drug Survival and Effectiveness

Understanding the risk factors associated with early discontinuation of drug treatment, should it be for poor tolerance, lack of efficacy or other reasons, allows us to take a step closer to personalised medicine. Several studies discussed below investigated factors associated with a higher risk of JAKi treatment discontinuation; most studies investigated baricitinib [60,62,74,82,85] and two considered baricitinib and tofacitinib [71,84].

Patients stratified by age (<65 years and ≥65 years) presented similar rates of baricitinib drug survival in ORBIT-RA [60], indicating no clear association between age and drug survival, whereas increased age was associated with higher drug survival in a UK-based (Southampton) study [85]. In the BSRBR-RA, during the first six months of treatment, there seemed to be minimal difference in drug survival between patients aged below or above 65 years but over time, older patients tended to discontinue the drug more frequently [29]. A single centre study by Deprez and colleagues [84] found that increased age, glucocorticoid usage at baseline and number of comorbidities were associated with an increased risk of JAKi discontinuation. The ORBIT-RA registry supports that a greater number of comorbidities is associated with a higher risk of baricitinib discontinuation [60] and a study of patients with RA in the Swiss Clinical Quality Management in Rheumatic Diseases Foundation (SCQM) register also identified that concomitant glucocorticoid use at baseline, as well as higher disease activity, is associated with a higher risk of JAKi treatment discontinuation [62]. In line with this, the Japanese TBCR-RA [74] showed that higher disease activity (measured by DAS28-CRP) was independently associated with lack of achievement of LDA (EULAR response criteria) with baricitinib. In the German Erlangen cohort, prior bDMARD use appeared to be associated with lower baricitinib continuation [82], a finding also seen in the BSRBR-RA [29], while a trend for an increased number of bDMARDs and lower continuation was observed in the ORBIT-RA study [60]. In the aforementioned Japanese registry, no previous b/tsDMARD use was independently associated with the achievement of EULAR LDA [74]. However, although high baseline HAQ-DI and a high number of prior bDMARDs were associated with JAKi (tofacitinib and baricitinib) discontinuation and resistance to JAKi treatment (the patient did not achieve LDA [CDAI score ≤2.8] after 24 weeks), these associations were not demonstrated for baricitinib alone in multivariate logistic regression analyses in the Japanese FIRST registry (pharmaco-resistance hazard ratio [HR] of 1.41; *p* = 0.09 [71]). In the IPS, the authors reported that bDMARD-naïve patients had greater improvements in CDAI scores compared with those who had an IR to bDMARDs, and as a potential consequence, a higher treatment discontinuation rate in the bDMARD-IR population was observed [64]. Most of these observations from RWE contrast with baricitinib RCT data that showed no impact of prior biologic usage on baricitinib efficacy [36]. Monotherapy showed similar drug survival as compared to combination therapy in SCQM-RA [63] and this was confirmed in the BSRBR-RA up to six months [29]; however, at 12 months follow-up in the BSRBR-RA, it seemed monotherapy was associated with higher drug survival, but this was not statistically tested [29]. By contrast, a retrospective, longitudinal cohort study of patient data from the Spanish ORBIT-RA registry showed that baricitinib drug survival was higher with combination therapy, although again no formal statistical analysis was performed [60].

In summary, high disease activity, concomitant glucocorticoids, increased number of previous b/tsDMARDs and greater number of comorbidities are generally associated with baricitinib treatment discontinuation. However, no consistent trends were observed with regard to an effect of age and concomitant csDMARDs on treatment discontinuation.

#### 3.2.2. Effectiveness of Baricitinib Monotherapy in RWE Settings

In the Swedish ARTIS study, baricitinib was as effective when used in monotherapy as in combination with a csDMARD, as highlighted by a good EULAR DAS28-ESR response, HAQ-DI improvement (defined as a decrease in HAQ-DI >0.2 at one-year evaluation compared with baseline) and CDAI REM (CDAI ≤2.8 at one-year evaluation) [65]. Monocentric studies in Erlangen, Germany, and Sapienza University of Rome, Italy, also found that baricitinib effectiveness was similar between monotherapy and combination therapy [78,82]. Additionally, a multicentre Italian study showed that compared with baricitinib monotherapy, the use of concomitant MTX was not associated with differences in REM (DAS28-CRP <2.6 and CDAI ≤2.8) and LDA (DAS28 ≥2.6 and ≤3.2 and CDAI ≤10) rates in both bDMARD-naïve and -IR patients [64]. These results of no differences in effectiveness between baricitinib monotherapy and combination therapy are consistent with those of two Japanese RWE studies, one that used DAS28-CRP data from the multicentre TBCR-RA [74] and a prospective, observational, multicentre study that used DAS28-ESR outcomes [75]. Finally, studies utilising Italian claims databases showed that csDMARDs could be tapered/discontinued in some baricitinib-treated patients [87,88]. In the analysis of data from patients with a diagnosis of RA during 2018, 69.8% of patients were using concomitant csDMARDs at baseline, which was reduced to 34.2% at follow-up, an effect most frequently observed in bDMARD-naïve patients [87]. In the analysis of data from patients with a diagnosis of RA during 2019, concomitant csDMARD use was 73.9% at baseline and 41.1% at follow-up; again, the reduction in csDMARD use was most pronounced in bDMARD-naïve patients [88].

#### 3.2.3. Effect of Baricitinib on Glucocorticoid Tapering

Italian studies and the multinational, prospective observational study, RA-BE-REAL, demonstrated that baricitinib allows a reduction of glucocorticoid use [64,77,78], which is in line with the latest EULAR recommendations [2]. Patients who are pharmaco-resistant are usually taking more glucocorticoids [99], and baricitinib allowed tapering of glucocorticoids in both bDMARD-naïve and bDMARD-IR patients. In an Italian administrative claims database, 87.9% of 149 baricitinib-treated patients were using concomitant glucocorticoids at baseline, a number that was almost halved at the follow-up visit when 45.7% of patients were still using glucocorticoids [87]. This finding was confirmed in a later analysis by the same authors in 445 baricitinib-treated patients [88]. Furthermore, two cohort studies (Italian and Japanese) also reported a reduction in glucocorticoid utilisation [74,83]. The Italian monocentre study at Careggi University Hospital in Florence, Italy showed that prednisone-equivalent was used at a mean daily dosage of 6.25 mg at baseline (with 74.4% of patients using glucocorticoids), which decreased from baseline to 2.75 mg after three months and to 1.86 mg after six months of treatment (*p* < 0.0001 for both) (with 40% patients remaining on glucocorticoids) [83]. The Japanese study from the TBCR-RA, showed that the dosage of prednisolone used (3.9 mg/week) decreased from baseline, when baricitinib was initiated, to 24 weeks (3.2 mg/week; *p* = 0.006) [74].

#### 3.2.4. Effect of Baricitinib on Ultrasound-Assessed Inflammation

Not only are clinical scores improved with baricitinib, but there is evidence to confirm that inflammatory processes are decreased in baricitinib-treated patients. Two Italian monocentric studies investigated the effect of baricitinib treatment on inflammation, as measured by ultrasound [78,83]. Tesei and colleagues (2021) [83] reported that synovitis and tenosynovitis analysed with greyscale or with power Doppler were significantly improved as early as at one month of treatment, while erosion scores remained unchanged throughout the six-month duration of the study, whereas Spinelli and colleagues (2021) [78] reported reductions in US inflammatory scores, reflecting the joint inflammatory status, as early as at one month of treatment. These observations were confirmed in another monocentric study, in Daegu, Republic of Korea [86], and are in line with the observed decrease in inflammatory biomarkers associated with joint destruction in RCTs of baricitinib [12].

#### 3.2.5. RWE Effectiveness and Drug Survival of Baricitinib Compared to TNFi and Other Mode of Action bDMARDs

Five real-world studies allowed comparison of the effectiveness of baricitinib versus TNFi treatment, four were register-based studies—SCQM-RA [62], ARTIS [65], DANBIO [66] and BIO-1 [61]—and one was a prospective observational study—RA-BE-REAL [77], that compared baricitinib with b/tsDMARDs (other than baricitinib). Table 2 shows that baricitinib-treated patients were generally older, with a longer disease duration and more previous treatments (statistical testing was not consistently reported). This suggests that despite EULAR recommendations to either use a TNFi or, after risk assessment, a JAKi in patients with an IR to csDMARD treatment [2], current practice is still to favour a TNFi as first line, and then baricitinib as second line or later in the treatment algorithm. With regard to effectiveness (see Table 3), CDAI LDA and REM rates at one year were similar between patients in the SCQM-RA treated with baricitinib and TNFis [62], whereas the ARTIS study reported greater improvements in CDAI and HAQ-DI after three months and one year in baricitinib- versus TNFi-treated patients [65]. Six-month interim analysis of data from RA-BE-REAL also suggested that a higher proportion of patients reached CDAI remission with baricitinib compared with b/tsDMARDs (25.6% vs. 18.5%) but no formal statistical testing was performed [77]. In these three studies and two other studies (Table 3), drug survival was consistently higher with baricitinib, especially after adjusting for confounding variables, which is critical when looking at unbalanced cohorts. Adjusted drug survival was higher for baricitinib versus TNFis in the Spanish BIO-1 registry in which all patients were bio-naïve [61].

Several other studies have investigated the effectiveness and drug survival of baricitinib versus bDMARDs and other tsDMARDs. In the FIRST registry in Japan, CDAI scores were lower for baricitinib versus tofacitinib after 24 weeks, at which time significantly higher rates of CDAI LDA and REM were observed in the baricitinib cohort. In this study, the risk of selection bias was reduced by use of the propensity score-based inverse probability treatment weighted method [71]. In the study from the ARTIS Study Group, baricitinib effectiveness, measured using DAS28-ESR, HAQ-DI and CDAI at three months and one year, was compared to that of abatacept, IL-6 inhibitors, rituximab and tofacitinib, and almost all comparisons showed at least similar, if not numerically or significantly higher, rates of effectiveness with baricitinib versus the other mode of action DMARDs; the exception was DAS28-ESR, which showed significantly better improvement and more ‘good’ EULAR responders with IL-6 inhibitors versus baricitinib at three months and one year, respectively [65]. Notably, the study from the ARTIS Study Group also reported that baricitinib as monotherapy was more effective for HAQ-DI improvement than abatacept, IL-6 inhibitors, rituximab, TNFis and tofacitinib used as monotherapy. Similar trends were also observed for CDAI REM rates, but differences were not significant, with the exception of the comparison with rituximab [65]. In the TBCR-RA, propensity score matched patients receiving baricitinib or tocilizumab generally had similar improvements in tender and swollen joint count and serum CRP levels, but PGA of disease activity was significantly more improved with baricitinib [73]. In addition, a significantly higher proportion of patients receiving baricitinib achieved Boolean REM at 24 weeks as compared to tocilizumab (33% vs. 15%, *p* = 0.027) [73]. These real-world findings support those of a matching-adjusted indirect comparison showing better efficacy of baricitinib versus tocilizumab in HAQ-DI score and VAS pain using data from RCTs [54]. Interestingly, when stratifying patients based on CRP decreases after 24 weeks of treatment, baricitinib, but not tocilizumab, was associated with improved PGA in patients who did not achieve target reductions in CRP levels [73]. Since pain is a dominant factor driving PGA [48,100], this observation appears to support the RCT analysis from Taylor and colleagues (2019) that reported an effect of baricitinib on pain irrespective of inflammation [56]. In the Kansai Consortium for Well-being of Rheumatic Disease Patients (ANSWER) registry of patients with RA in Japan, no difference was observed with regard to treatment discontinuation rates at 18 months between baricitinib, tofacitinib and sarilumab [70]. The lack of difference in drug survival between baricitinib and tofacitinib was also demonstrated in 24-week analyses from the FIRST registry in Japan [71]. However, the HRs for discontinuation for baricitinib were significantly lower compared with those for tofacitinib, IL-6 inhibitors and abatacept, but higher than those for rituximab, after adjusting for confounding variables in the SRQ registry in the study from the ARTIS Study Group [65]. In adjusted analyses, treatment discontinuation was significantly less likely with baricitinib than with adalimumab, tofacitinib, abatacept, certolizumab pegol, golimumab, sarilumab and infliximab in the study from the Danish DANBIO registry, and the numerical lower risk of discontinuation versus etanercept and tocilizumab did not reach significance; rituximab had a lower risk of discontinuation than baricitinib, but this difference was not significant [66].

Treatment of D2T RA is a great unmet need for clinicians. Analysis of data from 353 patients with D2T RA in the FIRST registry in Japan showed that JAKis significantly improved CDAI compared to TNFis after adjustment using the propensity-based inverse probability treatment weighted method whereas for IL-6 inhibitors and abatacept, CDAI improvement was not significantly different from that of TNFi. The HRs of severe AEs were comparable among the four treatment subgroups, JAKis, TNFis, IL-6 inhibitors and abatacept, in both D2T and b/tsDMARD-naïve patients [101].

#### 3.2.6. To Cycle or to Switch?

It is important to initiate a treatment change when RA is not sufficiently controlled since uncontrolled disease activity leads to an increased risk of comorbidities, including major adverse CV events (MACE) [102], VTE [103], malignancies [104] and serious infection [105]. EULAR recommendations for a treatment change allow for either cycling within a drug class or switching the mode of action of b/tsDMARDs [2]. After a TNFi-IR, both strategies have been shown to be valid [7,106,107,108,109]. However, an RCT showed that after a primary TNFi-IR, switching to a DMARD with another mode of action led to a higher percentage of patients attaining LDA at 24 weeks and one year as compared with patients who cycled to another TNFi (45% vs. 28% at 24 weeks; odds ratio [OR], 2.09; 95% confidence interval [CI], 1.27 to 3.43; *p* = 0.004 and 41% vs. 23% at 52 weeks; OR, 2.26; 95% CI, 1.33 to 3.86; *p* = 0.003) [110]. Another meta-analysis that included RCTs and observational studies confirmed that switching to a different mode of action DMARD after a TNFi failure is more effective and also associated with lower rates of withdrawal than cycling to a different TNFi [111]. Finally, patients from RA-BEAM initiated on treatment with adalimumab who were either rescued because of lack of efficacy or who were switched to baricitinib at entry into the long-term extension study showed improved disease responses (CDAI, SDAI, DAS28-ESR) after the switch [112]. On the other hand, patients who are JAKi-IR and cycle to another JAKi were shown to have higher drug survival than patients switched to bDMARDs in an adjusted analysis conducted on data from the SCQM-RA (JAKi vs. TNFi; HR 0.48; 95% CI 0.3 to 0.76) [91] as well as in a pooled international consortium of 17 RA registries, JAK-pot (JAKi vs. bDMARD HR 0.82; 95% CI 0.68 to 0.99; *p* = 0.04) [95]. Notably, in JAK-pot, it was observed that if the first JAKi was discontinued due to an AE, it was more likely that the second JAKi would also be stopped because of an AE; JAKi cyclers and TNFi switchers showed similar treatment effectiveness [95]. A Japanese study from the FIRST registry reported that JAKi cyclers were more likely than TNFi switchers to achieve CDAI LDA (64.3% vs. 18.8%) and REM (39.3% vs. 0%) [72].

### 3.3. Safety

Due to the chronic nature of RA, patients with high levels of inflammation are at risk of comorbidities including accelerated atherosclerosis [113], VTE [114], malignancy [115] and serious infection [116]. Hence, it is crucial to assess the long-term safety profile of baricitinib, and further studies are required to allow the differentiation between an adverse reaction due to a drug and a comorbidity due to the natural course of the disease.

#### 3.3.1. Baricitinib Safety in RCTs

**Figure 3:**
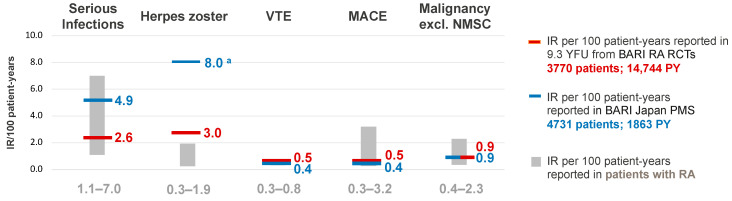
Rates of AEs of special interest with baricitinib in RCTs, a PMS study in Japan and in the general RA population [76,117,118,119,120]. ^a^ Herpes zoster incidence is higher in Asia compared to North America and Europe [121]. AEs, adverse events; BARI, baricitinib; excl., excluding; IR, incidence rate; MACE, major adverse cardiovascular events; NMSC, non-melanoma skin cancer; PMS, post-marketing surveillance; PY, patient-years; RA, rheumatoid arthritis; RCT, randomised controlled trial; VTE, venous thromboembolism; YFU, years follow-up.

In 2022, Taylor and colleagues reported on the largest integrated analysis database of baricitinib safety data (from nine Phase III/II/Ib clinical trials and one long-term extension study in RA) that included 3770 patients with RA and over 14,744 patient-years of baricitinib exposure [117]. These patients covered the clinical continuum of RA within the constraints of a clinical trial, and it was demonstrated that AEs were stable over time, with no dose response and no new safety risks observed with prolonged treatment [117]. Moreover, the observed incidence rates of AEs with baricitinib in the RA clinical trials were within the expected range for the typical RA population [118], with the exception of herpes zoster (Figure 3 [76,117,118,119,120,121]). The most common treatment emergent AEs reported during baricitinib treatment were nasopharyngitis, upper respiratory tract infections, bronchitis, urinary tract infections and herpes zoster [117]. Due to the JAK2 inhibitory effects of baricitinib, laboratory changes were also specifically considered in clinical trials. Investigations showed that changes in neutrophils, lymphocytes, platelets and haemoglobin were generally moderate and usually transient or reversible, infrequently resulting in permanent treatment discontinuation [122]. VTE was, however, considered an adverse drug reaction [9] because of the imbalance observed during the placebo-controlled period of RA-BEAM where six cases of VTE were observed in the baricitinib 4 mg group versus none in the placebo group [123]. Although in one meta-analysis of RCTs, JAKis were not reported to be associated with VTE during the placebo-controlled period [124], another meta-analysis showed that an association was seen at later timepoints [125]. It needs to be remembered when interpreting these data that clinical trials include a highly selected patient population and that the possibility of survivor bias—where people who do not respond to study treatment or who develop an AE would likely discontinue the study—could affect the results of longer-term analyses.

In a post hoc analysis of baricitinib data pooled from clinical trials and long-term extension studies in RA, it was demonstrated that patients with risk factors (one or more of: age ≥65 years, atherosclerotic CV disease [ASCVD], diabetes mellitus, hypertension, current smoking, high density lipoprotein cholesterol <40 mg/dL, body mass index [BMI] ≥30 kg/m^2^, poor mobility on EQ-5D or history of malignancy) showed an increased incidence of VTE, MACE, malignancy and serious infection as compared to patients without such risk factors [126], highlighting the need to be cautious when treating older patients with risk factors. When other indications for which baricitinib has been investigated are considered (systemic lupus erythematosus, alopecia areata, atopic dermatitis and COVID-19), incidence rates from RCTs for particular AEs are within the range expected for corresponding typical populations with those diseases [118].

#### 3.3.2. New EULAR Recommendations for Risk Management Regarding JAKi Safety

A meta-analysis of data from registries and administrative claims by Salinas and colleagues, discussed in more detail below, reported an increased risk of VTE with baricitinib versus TNFis (IR ratio [IRR] 1.51), and trends towards an increased risk of MACE (IRR 1.54) and serious infection (IRR 1.36) that did not reach significance [33]. Around the time when the results for the ORAL Surveillance [32] and the Salinas publication [33] were shared, several health authorities, including the EMA, reviewed all JAKi data and, based on data from these studies, required an update to the warnings and precautions for all marketed JAKis indicated for inflammatory diseases [127]. Of note, the US Food and Drug Administration (FDA) had already issued JAK-class box warnings in 2021 [128]. These warnings and precautions highlight that, for patients aged over 65 years, present or past smokers and patients presenting with risk factors for MACE, cancer, VTE and serious infections, JAKis should only be used if no suitable alternative treatment is available [127]. The ORAL Surveillance subgroup analysis by age showed an increased risk of malignancies and MACE in patients aged ≥65 years (vs. <65 years), and in these older patients, increased risk in those treated with tofacitinib versus TNFis [32]. In a post hoc analysis of ORAL Surveillance, it was shown that patients with a history of ASCVD are at increased risk of MACE with tofacitinib, whereas in those with no history of ASCVD, no increased risk relative to TNFis was observed [129]. Patients with, versus without, ASCVD were more likely to be male, aged ≥65 years, past smokers and have a history of diabetes mellitus, hypertension or hyperlipidaemia. Additionally, a separate post hoc analysis of ORAL Surveillance showed that the risk of MACE, VTE and non-serious infections excluding herpes zoster appeared higher for patients with active disease versus those in REM [130].

#### 3.3.3. Baricitinib Safety from RWE

Most of the RWE studies focusing on baricitinib only sporadically report AEs of special interest and no new safety signals have been identified; the AEs reported correspond to those observed in RCTs. However, this is based on limited data and no analysis involving incidence rates for a given rate of patient-years. The exception is the PMS study of baricitinib use in patients with RA from Japan (55% of patients were aged ≥65 years [mean 64 years]), which reported incidence rates for MACE, VTE and malignancies that were generally similar to those expected in an RA population; the rate of herpes zoster was higher than that expected in an RA population (Figure 3) [76]. This higher rate of herpes zoster with baricitinib versus the RA population has also been observed in RCTs.

#### 3.3.4. Baricitinib vs. TNFi Safety from RWE

RWE safety studies have the advantage of offering longer follow-up and allow comparisons between two or more drugs, but are without randomisation, which implies a higher risk of bias. It is known from the ARTIS Study Group that patients using non-TNFi biologics are older and have a higher disease activity and are less healthy (more likely to have a history of malignancy, serious infection, diabetes and other diseases) than those using TNFis, with the risk that non-TNFi biologics may appear more harmful than TNFis [131]. In line with this, we previously discussed that baricitinib is prescribed to patients who are generally older, with longer disease duration and who have more treatment failures as compared to those prescribed TNFis or b/tsDMARDs other than baricitinib, as highlighted by five studies that investigated effectiveness (statistical testing was not consistently reported) (Table 2 and Table 3) [61,62,65,66,77]. Furthermore, in a safety study of data from the French national health data system (Système National des Données de Santé [SNDS]) that compared JAKi users (tofacitinib and baricitinib) to TNFi users (adalimumab), JAKi users were older, with more treatment failure and had numerically higher rates of comorbidities versus TNFi users; however, no formal statistical testing of these data was performed [97]. A recent updated cohort study from the ARTIS Study Group SRQ registry also observed that TNFis are prescribed to patients with less treatment failure, lower disease activity and fewer comorbidities as compared to JAKis, but again no formal testing was performed [79]. An analysis from the JAK-pot study that included >9000 patients treated with JAKi and about 25,000 patients treated with TNFi had similar findings with respect to a higher number of prior b/tsDMARDs and longer disease duration and more comorbidities at JAKi treatment initiation, a finding again not tested statistically [94]. The aforementioned emphasises that patients treated with JAKi are different from patients treated with TNFi. There are statistical approaches available to account for confounding variables and these help to better compare two distinct populations; however, heterogeneity between models used presents advantages and disadvantages and can lead to contradictory results, as discussed here.

**Figure 4:**
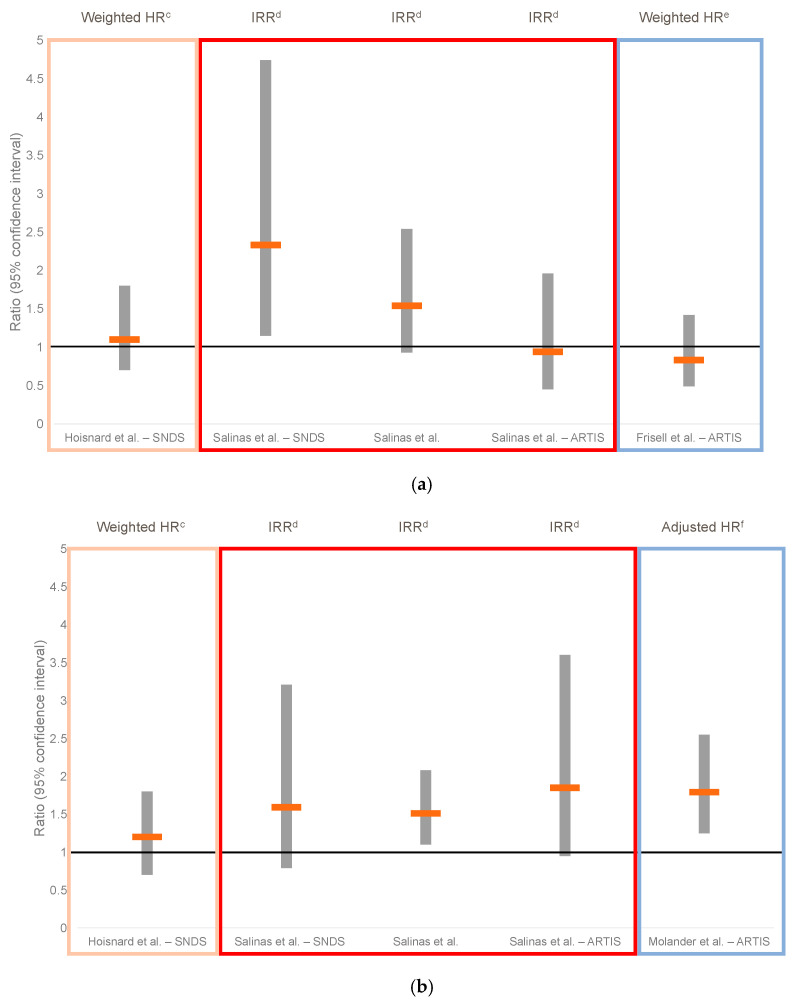
Differences in the reported risk of: (**a**) MACE; (**b**) VTE for baricitinib versus TNFis ^a^ in patients with RA according to different real-world analyses [33,79,80,97]. ^b^ Note that the reported data are from different analyses (weighted HR, adjusted HR and IRR) and therefore cannot be considered directly comparative. The side-by-side presentation is for illustrative purposes only. ^a^ All data are for baricitinib versus TNFis, with the exception of the ARTIS MACE analyses for which the comparator was the TNFi etanercept. ^b^ Table 4 shows the designs of the reported analyses. ^c^ Weighted using inverse probability of treatment weighting method and concomitant administration of MTX as a time-varying variable. Propensity score included age, sex, use of ≥1 csDMARD and use of bDMARD (within two years before index date and at index date), comorbidities (obesity, diabetes, COPD, severe chronic renal disease, dyslipidaemia, hypertension, atherosclerosis of extremities and history of CV or thromboembolic events within 10 years), use of systemic corticosteroids and NSAIDs within two years before index date and at index date, antiplatelet agents, anticoagulants. ^d^ Calculated after nearest-neighbour propensity score matching of treatment groups (baricitinib:TNFi) to balance baseline risk factors. Variables considered for inclusion in the propensity score models were risk factors specific to each outcome, including patient demographics, medical history (including comorbidities) and treatments for RA, where available from the original sources. ^e^ Weighted using baseline patient characteristics (age, sex, immigrant status, highest achieved education, rheumatoid factor/anti-citrullinated protein antibodies, RA duration, previous b/tsDMARD use, co-medication with conventional synthetic DMARDs and glucocorticosteroids, the 28-joint disease activity score, the Health Assessment Questionnaire-Disability Index, history of malignancy, infections, joint surgery, chronic pulmonary disease, diabetes, cardiovascular disease, depression and the sum of days hospitalized in last five years) from Cox regression adjusted with stabilised inverse probability of treatment weights constructed as the inverse of the predicted probability to have received the treatment actually received, multiplied by the sample proportion with the same treatment. ^f^ Estimated using Cox proportional hazards regression, adjusted for age, sex, line of therapy, comorbidities, socioeconomic variables, RA disease variables, civil status and smoking, using an indicator for missing variables. ARTIS, Anti-Rheumatic Therapy in Sweden; bDMARD, biologic disease-modifying antirheumatic drug; COPD, chronic obstructive pulmonary disease; csDMARD, conventional synthetic disease-modifying antirheumatic drug; CV, cardiovascular; HR, hazard ratio; IRR, incidence rate ratio; MACE, major adverse cardiovascular events; MTX, methotrexate; NSAIDs, non-steroidal anti-inflammatory drugs; RA, rheumatoid arthritis; SNDS, Système National des Données de Santé (French national health data system); TNFi, tumour necrosis factor inhibitor; tsDMARD, targeted-synthetic disease-modifying antirheumatic drug; VTE, venous thromboembolism.

Salinas and colleagues (2023) conducted a meta-analysis of real-world data from 14 post-marketing data sources including registries and administrative claims in Europe, the US and Japan [33]. Data regarding patients who experienced a VTE or MACE were mainly accessed from two sources: the Swedish ARTIS Study Group SRQ registry, which accounted for 39% of the total dataset, and a French national health data system (SNDS) that accounted for 32% of the total registry. The meta-analysis showed a trend toward an increased risk of MACE with baricitinib versus TNFis that was importantly driven by data from the SNDS administrative claims database; however, their analysis of data from the ARTIS registry did not show an increased risk. Interestingly, Hoisnard and colleagues (2023) [97] also analysed data from more than 15,000 patients in SNDS and used an alternative method to Salinas and colleagues (2023) [33] (Table 4; Figure 4). Whereas Salinas and colleagues (2023) [33] used propensity score matching to match patients from the two cohorts and remove the remaining patients from the analysis (16% of baricitinib patients), Hoisnard and colleagues (2023) [97] used propensity score weighting to assign patients’ outcomes a weight based on their baseline characteristics (age, sex, concomitant csDMARDs or bDMARDs, utilisation of non-steroidal anti-inflammatory drugs [NSAIDS] or glucocorticoids, comorbidities such as obesity and chronic obstructive pulmonary disease [COPD] and history of CV or thromboembolic events within 10 years). Both unweighted and weighted analyses by Hoisnard and colleagues revealed no increase in risk of MACE with baricitinib versus TNFis with a weighted HR of 1.1 [97]. This was also shown in sub-analyses of different populations, such as in patients aged ≥50 years with one CV risk factor, patients aged ≥65 years with one CV risk factor or patients with one CV risk factor. VTE risk assessed by Hoisnard and colleagues (2023) [97] before weighting matched that determined by Salinas and colleagues (2023) [33] and showed an increased risk of VTE with baricitinib; however, after weighting for covariables, the HR for VTE no longer showed an increased risk with baricitinib, in contrast to the findings of Salinas and colleagues (2023) [33].

Additional analyses of the ARTIS Study Group SRQ registry data, which unlike administrative claims data reports disease duration and activity, smoking status and medical history (including comorbidities and line of therapy), also assessed individual TNFis and compared them to other mode of action agents, including baricitinib [79,80] (Table 4; Figure 4). No increased risk of MACE with baricitinib compared with each TNFi individually was observed. Additionally, the discontinuation rate due to AEs for patients using baricitinib was significantly lower than that for etanercept (HR 0.57; 95% CI 0.45 to 0.73 vs. etanercept) and lower than all TNFis investigated in the study, although this was not tested statistically [79]. When compared with TNFis (as a class), the risk of VTE was increased with baricitinib [80]. Adjusting for covariables is of importance knowing that disease activity, smoking status and comorbidities will have an impact on the risk of MACE, VTE, malignancies and serious infection [102,103,104,105].

Although there are a few RWE studies of baricitinib that report conflicting results to those of Salinas and colleagues (2022) [33] with regard to MACE, two large, long-term post-marketing RCTs have been initiated to give more information about the risk of this particular event for baricitinib versus TNFi. RA-BRIDGE (NCT03915964) and RA-BRANCH (NCT04086745) are being conducted in >2500 and >1000 participants, respectively, with RA and ≥1 VTE risk factor with an IR or intolerance to ≥1 prior csDMARD or bDMARD. The studies will primarily compare the risk of VTE in patients treated with baricitinib versus TNFi, but are also planned to further inform rheumatologists about any MACE, cancer and serious infection risk for patients using baricitinib.

#### 3.3.5. JAKi Safety from RWE

Three RWE studies have shown an increased risk of VTE in patients treated with JAKis versus TNFis or no JAKi, with data from one registry study [80] and two administrative claims data sets, one of which was a self-controlled case using data from the SNDS [96,98]. In contrast, two other studies did not report an increased risk of VTE with JAKis: one US registry study comparing VTE in tofacitinib versus bDMARDs [132] and a population-based cohort study, also using data from the SNDS [97]. When MACE was considered, no association was reported with JAKi utilisation in the German RABBIT registry [68], in multivariate analyses of the Argentinian BIOBADASAR 3.0 registry [90] or in an analysis of the SNDS [97] as compared to TNFi users. Analysis of US registry data [132] and administrative claims data [133] did not report an association between MACE and tofacitinib utilisation compared with TNFi/bDMARD utilisation in the overall population, but a trend was observed in an ORAL Surveillance-duplicated cohort in the latter study [133].

Analysis of the Spanish registry, BIOBADASER 3.0, and the Argentinian registry, BIOBADASER 3.0, identified no increased risk of cancer in JAKi users compared with TNFi users [89,90]: a finding similar to that of a study using the SNDS administrative claims data [134]. Another study of administrative claims data investigating the association of tofacitinib and cancer led to similar observations [135]. Serious infections have not been reported to be associated with JAKis in the elderly [93], a finding supported by a study using administrative claims data in Korea; in this analysis, the risk of serious infections was not increased in a JAKi versus a TNFi cohort, with the exception of herpes zoster [136]. Finally, the recent publication from the ARTIS Study Group SRQ registry also showed no increased rate of serious infection with tofacitinib or baricitinib versus etanercept, but an increased rate of herpes zoster infection [79].

In the JAK-pot collaboration of 16 international RA registries with almost 35,000 patients, no increase in drug discontinuation rates due to AEs, a proxy for safety, was observed in the JAKi cohort compared with the TNFi cohort (TNFi vs. JAKi adjusted HR 1.11; 95% CI 0.98 to 1.25) [94].

#### 3.3.6. Risk Assessment for Personalised Medicine for Patients with RA

While it is thought that the future of RA management will be towards personalised medicine based on distinct phenotype and biomarkers [137], current risk mitigation strategies already allow for the prescription of a particular treatment to a specific patient. When TNFis were first used, tuberculosis (TB) was observed at a rate of almost 800 cases/100,000 patient-years in 2002, which dramatically reduced to 38 cases/100,000 patient-years in 2015 after HCPs implemented TB screening in daily practice [138]. Rheumatologists treating patients at risk of demyelination disease, cardiac insufficiency (Type III and IV) and bacterial or viral infections (TB, hepatitis B and opportunist infections) should be cautious when using TNFis [139,140,141,142,143]. IL-6 inhibitors should be avoided for patients with hepatic insufficiency and JAKis should be used with caution in patients aged over 65 years with CV, malignancy or VTE risk factors or recurrent serious infection. JAKis and abatacept have been reported to be effective and safe in patients with RA and interstitial lung disease [144].

Continued observational long-term studies will be required to assess and quantify the safety profile of baricitinib, especially real-world studies in which patients are older, have a longer disease duration and a greater likelihood of having a refractory condition [145]. New EMA warnings and precautions should have an impact on the way rheumatologists prescribe baricitinib. RA-BRIDGE and RA-BRANCH will help the scientific community understand whether the results seen in ORAL Surveillance, in particular those relating to MACE or malignancies, [32] are solely related to tofacitinib or rather, are a JAKi class effect.

## 4. Future Directions

Baricitinib has demonstrated rapid efficacy that is consistent in both DMARD pre-treated and naïve populations in clinical trials of RA and it has a favourable overall benefit:risk ratio, all of which have been thoroughly confirmed in the context of RWE. Moreover, baricitinib has been shown to be superior to TNFi in randomised clinical trials. In addition, a very recent pragmatic treat-to-target RWE study, which has the advantage of studying baricitinib in real-life but with a randomisation step, confirmed the superiority of baricitinib versus TNFi with regard to the proportion of patients reaching ACR50 at 12 weeks [146]. Furthermore, DAS28-CRP remission rates favoured baricitinib over TNFi at 12 weeks in this study (74% vs. 46%; *p* < 0.001). However, as in the case of many newly introduced therapies, baricitinib has tended to be used relatively late in the treatment paradigm. There is a compelling case for the earlier introduction of baricitinib in the treatment algorithm with a view to optimising achievable outcomes relating to symptoms and signs as well as patient-reported outcomes. In addition, use of baricitinib rather than a bDMARD as the first b/tsDMARD has been shown to result in lower costs and higher utility over a five-year period in patients with either moderate or severe RA and csDMARD-IR [147]. This approach would also minimise exposure to the drug by older patients with known CV risk factors, in whom the benefit:risk profile of baricitinib (and other JAKi) may be less favourable. If to be used in patients aged ≥65 years, those with moderate renal impairment or those at risk of AEs of special interest (CV events, malignancy, VTE and serious infections), a reduced dosage of baricitinib 2 mg/day is recommended with the possibility to step up to a higher baricitinib dose of 4 mg if RA is not controlled.

As knowledge grows with respect to the pathobiology of RA comorbidities and the relationship to particular mechanisms of action of drugs, it is anticipated that this will inform advances in a more personalised approach to treatment choice and improve the positioning of baricitinib. With indications in RA, alopecia areata, atopic dermatitis and COVID-19, baricitinib is now established as an effective treatment for controlling dysregulated immune responses.

## Figures and Tables

**Table 1:** Pharmacological characteristics of JAK inhibitors approved for the treatment of RA.

	Baricitinib	Tofacitinib	Upadacitinib	Filgotinib	Peficitinib
	[4,9,10,15]	[16,17,18]	[19,20,21]	[22,23,24]	[25,26]
Chemical structure	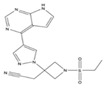	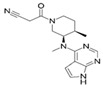	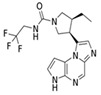	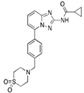	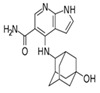
Dose	2 or 4 mg QD	5 mg BID	15 mg XR QD	200 mg QD	150 mg QD (100 mg QD ^a^)
		(5 mg QD ^a^)			
				(100 mg QD ^a^)	
		11 mg XR QD			
JAK selectivity (in vitro), IC_50_, nM	JAK1: 5.9,	JAK1: 3.2,	JAK1: 47,	JAK1: 10,	JAK1: 3.9,
	JAK2: 5.7,	JAK2: 4.1,	JAK2: 120,	JAK2: 28,	JAK2: 5.0,
	JAK3: 560,	JAK3: 1.6,	JAK3: 2304,	JAK3: 810,	JAK3: 0.7,
	TYK2: 53	TYK2: 34	TYK2: 4690	TYK2: 116	TYK2: 4.8
Half-life (hours)	12.5	~3	8–14	7 ^b^	10–18
Excretion	Parent and metabolites: 75% renal, 20% faeces	Unchanged parent: 70% hepatic, 30% renal	Parent and metabolites: 24% urine, 38% faeces	Parent and metabolites: 87% urine, 15% faeces	Parent and metabolites: ~37% urine, 57% faeces
Drug–drug interactions	Strong (OAT3) inhibitors	Strong CYP3A4 inhibitors or inducers, moderate CYP3A4 inhibitors with strong CYP2C19 inhibitors, immunosuppressants	Strong CYP3A4 inhibitors or inducers	CES2 inhibitors, CYP1A2 or P-gp or BCRP substrates	P-gp inhibitor verapamil

^a^ Selected populations. ^b^ The half-life of the active metabolite is 19 h. BCRP, breast cancer resistance protein; BID, twice daily; CES, carboxylesterase; CYP, cytochrome P450; IC_50_, half maximal inhibitory concentration; JAK, Janus kinase; OAT3, organic anion transporter-3; P-gp, P glycoprotein; QD, once daily; RA, rheumatoid arthritis; TYK, tyrosine kinase; XR, extended release.

**Table 2:** Baseline characteristics of baricitinib- and TNFi-treated RA patients in real-world studies.

	BIO-1 (Bio-naïve) [61]		SCQM-RA [62]		RA-BE-REAL [77]		ARTIS [65]		DANBIO [66]		
Patients, *n*	**BARI**	**TNFi**	**BARI ^a^**	**TNFi**	**BARI**	**b/tsDMARD ^b^**	**BARI**	**TNFi**	**BARI**	**ETN**	**ADA**
	63	33	273	408	509	565	1420	6036	275	1830	1332
Age (years)	60	56 ^c^	59	52 **	59	57 *	61	58 ^d^	59	57 ^d^	57 ^d^
Disease duration (years)	8 ^e^	7 ^d,e^	13	8 **	10	9 *	13	8 ^d^	14	10 ^d^	10 ^d^
Disease activity (CDAI)	NR	NR ^d^	15	14 ^c^	24	24 ^c^	20	18 ^d^	4.35 ^f^	3.75 ^d,f^	3.65 ^d,f^
Smokers (%)	NR	NR ^d^	NR	NR ^d^	NR	NR ^d^	11	12 ^d^	17	22 ^d^	23 ^d^
Bio-naïve (%)	100	100 ^d^	17	48 **^,g^	48	61 ^d^		Mostly ^d,h^	3	42 ^d^	55 ^d^
≥2 DMARDs (%)	NR	NR ^d^	63	29 **^,d,g^	39	29 ^d^	Mostly ^d,h^		86	26 ^d^	22 ^d^
Comorbidities (%)	NR	NR ^d^	NR	NR ^d^	NR	NR^d^	3 (VTE)	2 (VTE) ^d^	NR ^e^	NR ^d^	NR ^d^

^a^ Three-way ANOVA also compared BARI with other mode of action bDMARDs; statistical results are not presented for these analyses in this table. ^b^ Data in this cohort are from recipients of TNFi (*n* = 338), non-TNFi bDMARDs (*n* = 161) and non-baricitinib tsDMARDs (*n* = 66). ^c^ Not statistically significantly different from the TNFi or b/tsDMARD group. ^d^ Statistical analysis not reported/conducted. ^e^ Calculated by subtracting age at BIO-1 entry—age of diagnosis. ^f^ Disease Activity Score in 28 joints. ^g^ *p*-value was calculated for overall line of therapy. ^h^ Median line of therapy was third for the BARI group and first for the TNFi group. * *p* < 0.05 for BARI vs. TNFi or b/tsDMARD. ** *p* < 0.01 for BARI vs. TNFi or b/tsDMARD. ADA, adalimumab; ANOVA, analysis of variance; BARI, baricitinib; bDMARD, biologic disease-modifying antirheumatic drug; CDAI, Clinical Disease Activity Index; ETN, etanercept; *n*, number; NR, not reported; RA, rheumatoid arthritis; SD, standard deviation; TNFi, tumour necrosis factor inhibitor; SCQM, Swiss Clinical Quality Management in Rheumatic Diseases Foundation; tsDMARD, targeted synthetic disease-modifying antirheumatic drug; VTE, venous thromboembolism.

**Table 3:** Effectiveness and drug survival of baricitinib versus TNFi in real-world studies.

Real-World Study	Effectiveness		Survival (Kaplan–Meier)		Survival (Adjusted HR)	
			or % Discontinuation (Crude)			
	CDAI	DAS28-ESR	Overall	Bio-Naïve	Overall	Bio-Naïve
ARTIS [65]	BARI >> TNFi	BARI > TNFi	BARI = TNFi	NR	BARI >> TNFi	NR
	at 3 and	at 3 mo				
	12 mo					
SCQM-RA [62]	BARI = TNFi at 12 mo	NR	BARI > TNFi	BARI > TNFi	BARI >> TNFi	BARI >> TNFi
DANBIO [66]	NR	NR	BARI = TNFi	NR ^a^	BARI >> TNFi ^b^	NR ^a^
RA-BE-REAL [77]	BARI > b/tsDMARD ^c^ at 6 mo ^d^	NR	BARI > b/tsDMARD ^c^ at 6 mo ^d^	BARI > b/tsDMARD ^c^ at 6 mo ^d^	NR	NR
BIO-1 (Bio-naïve) [61]	NR	NR	NR	BARI > TNFi ^e^	NR	BARI >> TNFi

^a^ Insufficient BARI-treated patients were bio-naïve for analyses. ^b^ Statistically better than adalimumab, certolizumab, golimumab and infliximab. ^c^ Data in this cohort are from recipients of TNFi (*n* = 338), non-TNFi bDMARDs (*n* = 161) and non-BARI tsDMARDs (*n* = 66). ^d^ No statistical testing was performed. ^e^ *p* = 0.15 for discontinuation rate (no crude Kaplan–Meier analysis was reported). >, numerically but not significantly better than comparator (either not tested or significance not reached). >>, better than comparator as indicated by 95% CIs and/or <0.05. BARI, baricitinib; bDMARD, biologic disease-modifying antirheumatic drug; CDAI, Clinical Disease Activity Index; CI, confidence interval; DAS28-ESR, Disease Activity Score in 28 joints—erythrocyte sedimentation rate; HR, hazard ratio; mo, months; NR, not reported; SCQM-RA, Swiss Clinical Quality Management in Rheumatic Diseases Foundation—rheumatoid arthritis; TNFi, tumour necrosis factor inhibitor; tsDMARD, targeted synthetic disease-modifying antirheumatic drug.

**Table 4:** Design of real-world studies reporting differences in the reported risk of MACE with JAKi versus TNFi in RA ^a^.

	SNDS (Hoisnard et al., 2023) [97]	SNDS (Salinas et al., 2022) [33]	ARTIS (Salinas et al., 2022) [33]	ARTIS (Frisell et al., 2023; [79] Molander et al., 2023 [80])
Study design	Cohort study comparing new users of JAKis (tofacitinib or baricitinib) and TNFi (adalimumab)	Focused analysis on data from SNDS: reanalysis of data from the SNDS cohort study comparing new users of baricitinib and TNFi	Focused analysis on data from ARTIS: reanalysis of data from the ARTIS cohort study comparing new users of baricitinib and TNFi	Nationwide register-based cohort study comparing individual b/tsDMARDs (baricitinib vs. individual TNFi)
Study population	Eligible patients included in the SNDS data between 1 Jul 2017 and 31 May 2021	Eligible patients included in the SNDS data between 1 September 2017 and 31 December 2019	Eligible patients were identified between February 2017 and December 2020	All patients with RA in Sweden who started any b/tsDMARD between 1 January 2010 and 31 December 2020
Patients, *n*	8481 JAKi recipients (5065 baricitinib); 7354 TNFi recipients	2859 baricitinib recipients’ propensity score matched with TNFi recipients	1685 baricitinib recipients’ propensity score matched with TNFi patients	1837 baricitinib recipients; 20,117 b/tsDMARD recipients in total (MACE); 1825 baricitinib recipients; 19,950 TNFi recipients (VTE)
Exposure	JAKi median follow-up ^b^ 1.2 years in SNDS	Baricitinib median exposure 0.47 years in SNDS	Baricitinib mean exposure 1.38 years in ARTIS	Baricitinib mean exposure 1.9 years in ARTIS (MACE); NR (VTE)
Statistical analysis	Analysis results based on propensity score weighted (IPTW) Cox regression	Analysis based on propensity score matched cohorts evaluated with modified Poisson regression	Analysis based on propensity score matched cohorts evaluated with modified Poisson regression	Crude and adjusted incidence rates and Cox regressions (VTE) adjusted with stabilised IPTW (MACE)

^a^ Figure 4 shows the results of these analyses. ^b^ Follow-up was defined as the time to the occurrence of each event (MACE and VTE), death from any cause, exposure discontinuation or 31 December 2021, whichever came first. ARTIS, Anti-Rheumatic Therapy in Sweden; BARI, baricitinib; bDMARD, biologic disease-modifying antirheumatic drug; HR, hazard ratio; IPTW, inverse probability of treatment weighting; JAKi, Janus kinase inhibitor; MACE, major adverse cardiovascular events; NR, not reported; RA, rheumatoid arthritis; SNDS, Système National des Données de Santé (French national health data system); TNFi, tumour necrosis factor inhibitor; tsDMARD, targeted synthetic disease-modifying antirheumatic drug; VTE, venous thromboembolism.

## Data Availability

No new data were created or analysed in this study. Data sharing is not applicable to this article.

## Supplementary Materials

The following supporting information can be downloaded at: https://www.mdpi.com/article/10.3390/jcm12134527/s1, Table S1: Baseline demographics and characteristics of patients baricitinib-treated patients in real-world settings and RCTs.

## References

[1] Fraenkel L., Bathon J.M., England B.R., St Clair E.W., Arayssi T., Carandang K., Deane K.D., Genovese M., Huston K.K., Kerr G. (2021). 2021 American College of Rheumatology guideline for the treatment of rheumatoid arthritis. Arthritis Care Res..

[2] Smolen J.S., Landewé R.B.M., Bergstra S.A., Kerschbaumer A., Sepriano A., Aletaha D., Caporali R., Edwards C.J., Hyrich K.L., Pope J.E. (2022). EULAR recommendations for the management of rheumatoid arthritis with synthetic and biological disease-modifying antirheumatic drugs: 2022 update. Ann. Rheum. Dis..

[3] Tanaka Y., Luo Y., O’Shea J.J., Nakayamada S. (2022). Janus kinase-targeting therapies in rheumatology: A mechanisms-based approach. Nat. Rev. Rheumatol..

[4] Markham A. (2017). Baricitinib: First global approval. Drugs.

[5] Dougados M., van der Heijde D., Chen Y.C., Greenwald M., Drescher E., Liu J., Beattie S., Witt S., de la Torre I., Gaich C. (2017). Baricitinib in patients with inadequate response or intolerance to conventional synthetic DMARDs: Results from the RA-BUILD study. Ann. Rheum. Dis..

[6] Taylor P.C., Keystone E.C., van der Heijde D., Weinblatt M.E., Del Carmen Morales L., Reyes Gonzaga J., Yakushin S., Ishii T., Emoto K., Beattie S. (2017). Baricitinib versus placebo or adalimumab in rheumatoid arthritis. N. Engl. J. Med..

[7] Fleischmann R., Schiff M., van der Heijde D., Ramos-Remus C., Spindler A., Stanislav M., Zerbini C.A., Gurbuz S., Dickson C., de Bono S. (2017). Baricitinib, methotrexate, or combination in patients with rheumatoid arthritis and no or limited prior disease-modifying antirheumatic drug treatment. Arthritis Rheumatol..

[8] Genovese M.C., Kremer J., Zamani O., Ludivico C., Krogulec M., Xie L., Beattie S.D., Koch A.E., Cardillo T.E., Rooney T.P. (2016). Baricitinib in patients with refractory rheumatoid arthritis. N. Engl. J. Med..

[9] European Medicines Agency (2017). Summary of Product Characteristics, Olumiant (Baricitinib) Film-Coated Tablets: EU Summary of Product Characteristics. http://www.ema.europa.eu/en/documents/product-information/olumiant-epar-product-information_en.pdf.

[10] (2022). OLUMIANT (Baricitinib): US Prescribing Information. https://www.accessdata.fda.gov/drugsatfda_docs/label/2022/207924s006lbl.pdf.

[11] McInnes I.B., Byers N.L., Higgs R.E., Lee J., Macias W.L., Na S., Ortmann R.A., Rocha G., Rooney T.P., Wehrman T. (2019). Comparison of baricitinib, upadacitinib, and tofacitinib mediated regulation of cytokine signaling in human leukocyte subpopulations. Arthritis Res. Ther..

[12] Thudium C.S., Bay-Jensen A.C., Cahya S., Dow E.R., Karsdal M.A., Koch A.E., Zhang W., Benschop R.J. (2020). The Janus kinase 1/2 inhibitor baricitinib reduces biomarkers of joint destruction in moderate to severe rheumatoid arthritis. Arthritis Res. Ther..

[13] Adam S., Simon N., Steffen U., Andes F.T., Scholtysek C., Müller D.I.H., Weidner D., Andreev D., Kleyer A., Culemann S. (2020). JAK inhibition increases bone mass in steady-state conditions and ameliorates pathological bone loss by stimulating osteoblast function. Sci. Transl. Med..

[14] Simon D., Minopoulou I., Kemenes S., Bayat S., Tascilar K., Mutlu M.Y., Valor-Méndez L., Krönke G., Hueber A.J., Schett G. (2023). Baricitinib improves bone properties and biomechanics in patients with rheumatoid arthritis—Results of the prospective interventional BARE BONE trial. Arthritis Rheumatol..

[15] Fridman J.S., Scherle P.A., Collins R., Burn T.C., Li Y., Li J., Covington M.B., Thomas B., Collier P., Favata M.F. (2010). Selective inhibition of JAK1 and JAK2 is efficacious in rodent models of arthritis: Preclinical characterization of INCB028050. J. Immunol..

[16] European Medicines Agency Summary of Product Characteristics, Xeljanz, INN-Tofacitinib Citrate. https://www.ema.europa.eu/en/documents/product-information/xeljanz-epar-product-information_en.pdf.

[17] (2018). XELJANZ (Tofacitinib): US Prescribing Information. https://www.accessdata.fda.gov/drugsatfda_docs/label/2018/203214s018lbl.pdf.

[18] Meyer D.M., Jesson M.I., Li X., Elrick M.M., Funckes-Shippy C.L., Warner J.D., Gross C.J., Dowty M.E., Ramaiah S.K., Hirsch J.L. (2010). Anti-inflammatory activity and neutrophil reductions mediated by the JAK1/JAK3 inhibitor, CP–690,550, in rat adjuvant-induced arthritis. J. Inflamm..

[19] European Medicines Agency Summary of Product Characteristics, Rinvoq, INN-Upadacitinib. https://www.ema.europa.eu/en/documents/product-information/rinvoq-epar-product-information_en.pdf.

[20] (2019). RINVOQ (Upadacitinib): US Prescribing Information. https://www.accessdata.fda.gov/drugsatfda_docs/label/2022/211675s003lbl.pdf.

[21] Parmentier J.M., Voss J., Graff C., Schwartz A., Argiriadi M., Friedman M., Camp H.S., Padley R.J., George J.S., Hyland D. (2018). In vitro and in vivo characterization of the JAK1 selectivity of upadacitinib (ABT-494). BMC Rheumatol..

[22] European Medicines Agency Summary of Product Characteristics, Jyseleca, INN-Filgotinib. https://www.ema.europa.eu/en/documents/product-information/jyseleca-epar-product-information_en.pdf.

[23] Van Rompaey L., Galien R., van der Aar E.M., Clement-Lacroix P., Nelles L., Smets B., Lepescheux L., Christophe T., Conrath K., Vandeghinste N. (2013). Preclinical characterization of GLPG0634, a selective inhibitor of JAK1, for the treatment of inflammatory diseases. J. Immunol..

[24] Angelini J., Talotta R., Roncato R., Fornasier G., Barbiero G., Dal Cin L., Brancati S., Scaglione F. (2020). JAK-Inhibitors for the Treatment of Rheumatoid Arthritis: A Focus on the Present and an Outlook on the Future. Biomolecules.

[25] Astellas Pharma Smyraf® Tablets 50 mg and 100 mg, Peficitinib Hydrobromide: Review Report. https://www.pmda.go.jp/files/000233074.pdf.

[26] Markham A., Keam S.J. (2019). Peficitinib: First global approval. Drugs.

[27] Posada M.M., Cannady E.A., Payne C.D., Zhang X., Bacon J.A., Pak Y.A., Higgins J.W., Shahri N., Hall S.D., Hillgren K.M. (2017). Prediction of transporter-mediated drug-drug interactions for baricitinib. Clin. Transl. Sci..

[28] Emery P., Tanaka Y., Cardillo T., Schlichting D., Rooney T., Beattie S., Helt C., Smolen J.S. (2020). Temporary interruption of baricitinib: Characterization of interruptions and effect on clinical outcomes in patients with rheumatoid arthritis. Arthritis Res. Ther..

[29] Edwards C.J., Mount J., Meeks A., Gulati T., Zaremba-Pechmann L., Sheesh M., Larsson E., Dennison E. (2023). Characteristics of patients initiating treatment with baricitinib and outcomes at follow-up: Analysis of BSRBR-RA registry data. Rheumatology.

[30] Fleischmann R., Alam J., Arora V., Bradley J., Schlichting D.E., Muram D., Smolen J.S. (2017). Safety and efficacy of baricitinib in elderly patients with rheumatoid arthritis. RMD Open.

[31] European Medicines Agency Meeting Highlights from the Pharmacovigilance Risk Assessment Committee (PRAC) 9–12 January 2023. https://www.ema.europa.eu/en/news/meeting-highlights-pharmacovigilance-risk-assessment-committee-prac-9-12-january-2023.

[32] Ytterberg S.R., Bhatt D.L., Mikuls T.R., Koch G.G., Fleischmann R., Rivas J.L., Germino R., Menon S., Sun Y., Wang C. (2022). Cardiovascular and cancer risk with tofacitinib in rheumatoid arthritis. N. Engl. J. Med..

[33] Salinas C.A., Louder A., Polinski J., Zhang T.C., Bower H., Phillips S., Song Y., Rashidi E., Bosan R., Chang H.C. (2023). Evaluation of VTE, MACE, and serious infections among patients with RA treated with baricitinib compared to TNFi: A multi-database study of patients in routine care using disease registries and claims databases. Rheumatol. Ther..

[34] Yang Y., Li X.F., Zhang X., Bao C.D., Hu J.K., Xu J.H., Li X.P., Xu J., He D.Y., Li Z.J. (2020). Efficacy and safety of baricitinib in Chinese rheumatoid arthritis patients and the subgroup analyses: Results from study RA-BALANCE. Rheumatol. Ther..

[35] Li Z.G., Hu J.K., Li X.P., Yang Y., Li X.F., Xu J.H., Zhang X., Xu J., Bao C.D., He D.Y. (2021). Rapid onset of efficacy of baricitinib in Chinese patients with moderate to severe rheumatoid arthritis: Results from study RA-BALANCE. Adv. Ther..

[36] Genovese M.C., Kremer J.M., Kartman C.E., Schlichting D.E., Xie L., Carmack T., Pantojas C., Sanchez Burson J., Tony H.P., Macias W.L. (2018). Response to baricitinib based on prior biologic use in patients with refractory rheumatoid arthritis. Rheumatology.

[37] Caporali R., Aletaha D., Sanmartí R., Takeuchi T., Mo D., Haladyj E., Zaremba-Pechmann L., Taylor P.C. (2022). POS0701 Long-term efficacy of baricitinib in patients with rheumatoid arthritis who have had inadequate response to csDMARDs: Results from RA-beyond up to 7 years of treatment. Ann. Rheum. Dis..

[38] Caporali R., Aletaha D., Sanmartí R., Takeuchi T., Mo D., Haladyj E., Zaremba-Pechmann L., Taylor P.C. (2022). POS0682 Long-term efficacy of baricitinib in patients with rheumatoid arthritis with inadeqaute response to bDMARDs: Results from RA-beyond following 6.9 years of treatment. Ann. Rheum. Dis..

[39] Fleischmann R., Pangan A.L., Song I.H., Mysler E., Bessette L., Peterfy C., Durez P., Ostor A.J., Li Y., Zhou Y. (2019). Upadacitinib Versus Placebo or Adalimumab in Patients with Rheumatoid Arthritis and an Inadequate Response to Methotrexate: Results of a Phase III, Double-Blind, Randomized Controlled Trial. Arthritis Rheumatol..

[40] Fleischmann R., Mysler E., Hall S., Kivitz A.J., Moots R.J., Luo Z., DeMasi R., Soma K., Zhang R., Takiya L. (2017). Efficacy and safety of tofacitinib monotherapy, tofacitinib with methotrexate, and adalimumab with methotrexate in patients with rheumatoid arthritis (ORAL Strategy): A phase 3b/4, double-blind, head-to-head, randomised controlled trial. Lancet.

[41] Combe B., Kivitz A., Tanaka Y., van der Heijde D., Simon J.A., Baraf H.S.B., Kumar U., Matzkies F., Bartok B., Ye L. (2021). Filgotinib versus placebo or adalimumab in patients with rheumatoid arthritis and inadequate response to methotrexate: A phase III randomised clinical trial. Ann. Rheum. Dis..

[42] van der Heijde D., Durez P., Schett G., Naredo E., Østergaard M., Meszaros G., De Leonardis F., de la Torre I., López-Romero P., Schlichting D. (2018). Structural damage progression in patients with early rheumatoid arthritis treated with methotrexate, baricitinib, or baricitinib plus methotrexate based on clinical response in the phase 3 RA-BEGIN study. Clin. Rheumatol..

[43] López-Romero P., de la Torre I., Haladyj E., Aletaha D., Smolen J.S. (2022). Baricitinib further enhances disease-modifying effects by uncoupling the link between disease activity and joint structural progression in patients with rheumatoid arthritis. Ann. Rheum. Dis..

[44] Smolen J.S., Han C., van der Heijde D.M., Emery P., Bathon J.M., Keystone E., Maini R.N., Kalden J.R., Aletaha D., Baker D. (2009). Radiographic changes in rheumatoid arthritis patients attaining different disease activity states with methotrexate monotherapy and infliximab plus methotrexate: The impacts of remission and tumour necrosis factor blockade. Ann. Rheum. Dis..

[45] Smolen J.S., Avila J.C.M., Aletaha D. (2012). Tocilizumab inhibits progression of joint damage in rheumatoid arthritis irrespective of its anti-inflammatory effects: Disassociation of the link between inflammation and destruction. Ann. Rheum. Dis..

[46] Aletaha D., Alasti F., Smolen J.S. (2013). Rituximab dissociates the tight link between disease activity and joint damage in rheumatoid arthritis patients. Ann. Rheum. Dis..

[47] van der Heijde D., Kartman C.E., Xie L., Beattie S., Schlichting D., Mo D., Durez P., Tanaka Y., Fleischmann R. (2022). Radiographic progression of structural joint damage over 5 years of baricitinib treatment in patients with rheumatoid arthritis: Results from RA-BEYOND. J. Rheumatol..

[48] Khan N.A., Spencer H.J., Abda E., Aggarwal A., Alten R., Ancuta C., Andersone D., Bergman M., Craig-Muller J., Detert J. (2012). Determinants of discordance in patients’ and physicians’ rating of rheumatoid arthritis disease activity. Arthritis Care Res..

[49] van der Elst K., Meyfroidt S., De Cock D., De Groef A., Binnard E., Moons P., Verschueren P., Westhovens R. (2016). Unraveling patient-preferred health and treatment outcomes in early rheumatoid arthritis: A longitudinal qualitative study. Arthritis Care Res..

[50] Lee Y.C., Nassikas N.J., Clauw D.J. (2011). The role of the central nervous system in the generation and maintenance of chronic pain in rheumatoid arthritis, osteoarthritis and fibromyalgia. Arthritis Res. Ther..

[51] Ishida M., Kuroiwa Y., Yoshida E., Sato M., Krupa D., Henry N., Ikeda K., Kaneko Y. (2018). Residual symptoms and disease burden among patients with rheumatoid arthritis in remission or low disease activity: A systematic literature review. Mod. Rheumatol..

[52] Rifbjerg-Madsen S., Christensen A.W., Christensen R., Hetland M.L., Bliddal H., Kristensen L.E., Danneskiold-Samsøe B., Amris K. (2017). Pain and pain mechanisms in patients with inflammatory arthritis: A Danish nationwide cross-sectional DANBIO registry survey. PLoS ONE.

[53] Taylor P.C., Alten R., Álvaro Gracia J.M., Kaneko Y., Walls C., Quebe A., Jia B., Bello N., Terres J.R., Fleischmann R. (2022). Achieving pain control in early rheumatoid arthritis with baricitinib monotherapy or in combination with methotrexate versus methotrexate monotherapy. RMD Open.

[54] Fautrel B., Zhu B., Taylor P.C., van de Laar M., Emery P., De Leonardis F., Kannowski C.L., Nicolay C., Kadziola Z., De La Torre I. (2020). Comparative effectiveness of improvement in pain and physical function for baricitinib versus adalimumab, tocilizumab and tofacitinib monotherapies in rheumatoid arthritis patients who are naïve to treatment with biologic or conventional synthetic disease-modifying antirheumatic drugs: A matching-adjusted indirect comparison. RMD Open.

[55] Michaud K., Pope J.E., Emery P., Zhu B., Gaich C.L., DeLozier A.M., Zhang X., Dickson C.L., Smolen J.S. (2019). Relative impact of pain and fatigue on work productivity in patients with rheumatoid arthritis from the RA-BEAM baricitinib trial. Rheumatol. Ther..

[56] Taylor P.C., Lee Y.C., Fleischmann R., Takeuchi T., Perkins E.L., Fautrel B., Zhu B., Quebe A.K., Gaich C.L., Zhang X. (2019). Achieving pain control in rheumatoid arthritis with baricitinib or adalimumab plus methotrexate: Results from the RA-BEAM trial. J. Clin. Med..

[57] Fautrel B., Kirkham B., Pope J.E., Takeuchi T., Gaich C., Quebe A., Zhu B., de la Torre I., De Leonardis F., Taylor P.C. (2019). Effect of baricitinib and adalimumab in reducing pain and improving function in patients with rheumatoid arthritis in low disease activity: Exploratory analyses from RA-BEAM. J. Clin. Med..

[58] Smolen J.S., Kremer J.M., Gaich C.L., DeLozier A.M., Schlichting D.E., Xie L., Stoykov I., Rooney T., Bird P., Sánchez Bursón J.M. (2017). Patient-reported outcomes from a randomised phase III study of baricitinib in patients with rheumatoid arthritis and an inadequate response to biological agents (RA-BEACON). Ann. Rheum. Dis..

[59] Takeuchi T., Genovese M.C., Haraoui B., Li Z., Xie L., Klar R., Pinto-Correia A., Otawa S., López-Romero P., de la Torre I. (2019). Dose reduction of baricitinib in patients with rheumatoid arthritis achieving sustained disease control: Results of a prospective study. Ann. Rheum. Dis..

[60] Hernández-Cruz B., Rosas J., Díaz-Torné C., Belzunegui J., García-Vicuña R., Inciarte-Mundo J., Pons A., Millán A.M., Jeria-Navarro S., Valero J.A. (2022). Real-world treatment patterns and clinical outcomes of baricitinib in rheumatoid arthritis patients in Spain: Results of a multicenter, observational study in routine clinical practice (The ORBIT-RA study). Rheumatol. Ther..

[61] Rosas J., Pons A., Barber X., Seabre-Gallego J.M., Santos Soler G., Soler-Giner E., Bernal J.A., Raga A., Raya-Santos C., Cortés-Quiroz J.C. (2022). POS0657 Survival of baricitinib vs anti-TNF as the first biological drug in patients with rheumatoid arthritis, in clinical practice: Results of a local registry. Ann. Rheum. Dis..

[62] Gilbert B., Courvoisier D., Mongin D., Lauper K., Perrier C., Müller R., Finckh A. (2021). POS0668 Real-world effectiveness of baricitinib in the Swiss rheumatoid arthiritis register (SCQM-RA). Ann. Rheum. Dis..

[63] Gilbert B., Mongin D., Nham E., Courvoisier D., Lauper K., Laedermann C., Müller R., Finckh A. (2022). POS0435 Impact of combination therapy with csDMARDs on the effectiveness of biologic or targeted synthetic DMARDs in a real-life setting: Results from the Swiss rheumatoid arthritis register (SCQM-RA). Ann. Rheum. Dis..

[64] Guidelli G.M., Viapiana O., Luciano N., De Santis M., Boffini N., Quartuccio L., Birra D., Conticini E., Chimenti M.S., Bazzani C. (2021). Efficacy and safety of baricitinib in 446 patients with rheumatoid arthritis: A real-life multicentre study. Clin. Exp. Rheumatol..

[65] Barbulescu A., Askling J., Chatzidionysiou K., Forsblad-d’Elia H., Kastbom A., Lindström U., Turesson C., Frisell T. (2022). Effectiveness of baricitinib and tofacitinib compared with bDMARDs in RA: Results from a cohort study using nationwide Swedish register data. Rheumatology.

[66] Egeberg A., Rosenø N.A.L., Aagaard D., Lørup E.H., Nielsen M.L., Nymand L., Kristensen L.E., Thyssen J.P., Thomsen S.F., Cordtz R.L. (2022). Drug survival of biologics and novel immunomodulators for rheumatoid arthritis, axial spondyloarthritis, psoriatic arthritis, and psoriasis—A nationwide cohort study from the DANBIO and DERMBIO registries. Sem. Arthritis Rheum..

[67] Meissner Y., Kekow J., Klopsch T., Kühne C., Zink A., Strangfeld A. Erste Erfahrungen mit baricitinib aus dem rheumatologischen alltag. RA.05. Kongress der Deutschen Gesellschaft für Rheumatologie (DGRh). Proceedings of the Congress Center Rosengarten.

[68] Meissner Y., Albrecht K., Kekow J., Zinke S., Tony H.P., Schaefer M., Strangfeld A. (2022). OP0135 Risk of cardiovascular events under Janus kinase inhibitors in patients with rheumatoid arthririts: Observational data from the German RABBIT register. Ann. Rheum. Dis..

[69] Leeb B.F., Spellitz F., Eichbauer-Sturm G., Herold M., Stetter M., Puchner R., St. Singer F., Fritsch-Stork R. (2021). Januskinase inhibitors to treat rheumatoid arthritis: Real world data match clinical trial results an evaluation by BioReg, the Austrian registry for biologicals, biosimilars, and targeted synthetic DMARDS in the treatment of inflammatory rheumatic diseases. Rheumatol. Curr. Res..

[70] Ebina K., Hirano T., Maeda Y., Yamamoto W., Hashimoto M., Murata K., Onishi A., Jinno S., Hara R., Son Y. (2021). Drug retention of sarilumab, baricitinib, and tofacitinib in patients with rheumatoid arthritis: The ANSWER cohort study. Clin. Rheumatol..

[71] Miyazaki Y., Nakano K., Nakayamada S., Kubo S., Inoue Y., Fujino Y., Tanaka Y. (2021). Efficacy and safety of tofacitinib versus baricitinib in patients with rheumatoid arthritis in real clinical practice: Analyses with propensity score-based inverse probability of treatment weighting. Ann. Rheum. Dis..

[72] Miyazaki Y., Nakayamada S., Kubo S., Sonomoto K., Inoue Y., Fukuyo S., Hanami K., Tanaka Y. (2022). Characteristics and treatment-selection in patients with rheumatoid arthritis and with inadequate response to Janus kinase inhibitors. Arthritis Rheumatol..

[73] Asai S., Takahashi N., Kobayakawa T., Kaneko A., Watanabe T., Kato T., Nishiume T., Ishikawa H., Yoshioka Y., Kanayama Y. (2021). Comparison of the effects of baricitinib and tocilizumab on disease activity in patients with rheumatoid arthritis: A propensity score matching analysis. Clin. Rheumatol..

[74] Takahashi N., Asai S., Kobayakawa T., Kaneko A., Watanabe T., Kato T., Nishiume T., Ishikawa H., Yoshioka Y., Kanayama Y. (2020). Predictors for clinical effectiveness of baricitinib in rheumatoid arthritis patients in routine clinical practice: Data from a Japanese multicenter registry. Sci. Rep..

[75] Iwamoto N., Sato S., Kurushima S., Michitsuji T., Nishihata S., Okamoto M., Tsuji Y., Endo Y., Shimizu T., Sumiyoshi R. (2021). Real-world comparative effectiveness and safety of tofacitinib and baricitinib in patients with rheumatoid arthritis. Arthritis Res. Ther..

[76] Takagi M., Atsumi T., Matsuno H., Tamura N., Fujii T., Okamoto N., Takahashi N., Nakajima A., Nakajima A., Tsujimoto N. (2022). Safety and effectiveness of baricitinib for rheumatoid arthritis in Japanese clinical practice: 24-week results of all-case post-marketing surveillance. Mod. Rheumatol..

[77] Alten R., Burmester G.R., Matucci-Cerinic M., Salmon J.H., López-Romero P., Fakhouri W., de la Torre I., Zaremba-Pechmann L., Holzkämper T., Fautrel B. (2023). The RA-BE-REAL multinational, prospective, observational study in patients with rheumatoid arthritis receiving baricitinib, targeted synthetic, or biologic disease-modifying therapies: A 6-month interim analysis. Rheumatol. Ther..

[78] Spinelli F.R., Ceccarelli F., Garufi C., Duca I., Mancuso S., Cipriano E., Dell’Unto E., Alessandri C., Di Franco M., Perricone C. (2021). Effectiveness and safety of baricitinib in rheumatoid arthritis: A monocentric, longitudinal, real-life experience. Clin. Exp. Rheumatol..

[79] Frisell T., Bower H., Morin M., Baecklund E., Di Giuseppe D., Delcoigne B., Feltelius N., Forsblad-d’Elia H., Lindqvist E., Lindström U. (2023). Safety of biological and targeted synthetic disease-modifying antirheumatic drugs for rheumatoid arthritis as used in clinical practice: Results from the ARTIS programme. Ann. Rheum. Dis..

[80] Molander V., Bower H., Frisell T., Delcoigne B., Di Giuseppe D., Askling J., ARTIS Study Group (2023). Venous thromboembolism with JAK inhibitors and other immune-modulatory drugs: A Swedish comparative safety study among patients with rheumatoid arthritis. Ann. Rheum. Dis..

[81] Fitton J., Melville A.R., Emery P., Nam J.L., Buch M.H. (2021). Real-world single centre use of JAK inhibitors across the rheumatoid arthritis pathway. Rheumatology.

[82] Bayat S., Tascilar K., Bohr D., Krönke G., Simon D., Knitza J., Hartmann F., Schett G., Kleyer A. (2022). Efficacy and drug persistence of baricitinib monotherapy is similar to combination therapy in patients with active RA: A prospective observational study. RMD Open.

[83] Tesei G., Cometi L., Nacci F., Terenzi R., Tofani L., Capassoni M., Bartoli F., Fiori G., Matucci-Cerinic M., Bruni C. (2021). Baricitinib in the treatment of rheumatoid arthritis: Clinical and ultrasound evaluation of a real-life single-centre experience. Ther. Adv. Musculoskelet. Dis..

[84] Deprez V., Le Monnier L., Sobhy-Danial J.M., Grados F., Henry-Desailly I., Salomon-Goëb S., Rabin T., Ristic S., Fumery M., Fardellone P. (2020). Therapeutic maintenance of baricitinib and tofacitinib in real life. J. Clin. Med..

[85] Lwin M.N., Holroyd C., Edwards C.J. (2021). O10 Characteristics of patients who discontinued baricitinib treatment within 12 months and reasons for discontinuation: Real-world data. Rheumatology.

[86] Kim S.K., Jung U.H., Kim J.W., Choe J.Y. (2021). The beneficial effect of baricitinib on ultrasound-detected synovial inflammation and bone damage in rheumatoid arthritis: Preliminarily data from single center-based observational study for 24 weeks. Medicine.

[87] Perrone V., Losi S., Rogai V., Antonelli S., Fakhouri W., Giovannitti M., Giacomini E., Sangiorgi D., Degli Esposti L. (2020). Real-world analysis of therapeutic patterns in patients affected by rheumatoid arthritis in Italy: A focus on baricitinib. Rheumatol. Ther..

[88] Perrone V., Losi S., Rogai V., Antonelli S., Fakhouri W., Giovannitti M., Giacomini E., Sangiorgi D., Degli Esposti L. (2021). Treatment patterns and pharmacoutilization in patients affected by rheumatoid arthritis in Italian settings. Int. J. Environ. Res. Public Health.

[89] Castrejon I., Molina Collada J., Perez-garcia C., Vela-Casasempere P., Díaz-Torné C., Bohórquez C., Blanco J.M., Sánchez-Alonso F., on behalf of BIOBADASER (2022). POS1439 Cancer in patients with rheumatic diseases exposed to different biologic and targeted synthetic DMARDS in real-world clinical practice: Data from a multicenter register. Ann. Rheum. Dis..

[90] Brigante A., Quintana R., Isnardi C., Roberts K., Gomez G., Haye Salinas M., Soriano E., Pons-Estel G., De la Vega M., Kerzberg O. (2022). Cardiovascular and oncologic outcomes of anti-TNF alfa and JAK inhibitors in patients with rheumatoid and psoriatic arthritis. Real world data and insights of BIOBADASAR 3.0 registry. Arthritis Rheumatol..

[91] Amstad A., Papagiannoulis E., Scherer A., Rubbert-Roth A., Finckh A., Mueller R., Dudler J., Möller B., Villiger P.M., Schulz M.M.P. (2022). Comparison of drug retention of TNF inhibitors, other biologics and JAK inhibitors in RA patients who discontinued JAK inhibitor therapy. Rheumatology.

[92] Ciciriello S., Smith T., O’Sullivan C., Tymms K., Youssef P., Mathers D., Deakin C., Griffiths H., Littlejohn G. (2021). POS0223 Patterns of Janus kinase inhibitor cycling for the management of rheumatoid arthritis in real-world clinical practice: An analysis of the OPAL dataset. Ann. Rheum. Dis..

[93] Strangfeld A., Manger B., Worsch M., Schmeiser T., Zink A., Schaefer M. (2021). OP0116 Elderly patients are not at increased risk of serious infections when receiving bDMARDS or JAK inhibitors compared to csDMARD treatment. Ann. Rheum. Dis..

[94] Lauper K., Aymon R., Mongin D., Bergstra S.A., Choquette D., Codreanu C., Cordtz R., De Cock D., Dreyer L., Elkayam O. (2022). Evaluation of treatment discontinuation due to adverse events, and the effect of cardiovascular risk factors or type of JAK-inhibitors: An international collaboration of registries of rheumatoid arthritis patients (the ‘JAK-pot‘ study) [abstract]. Arthritis Rheumatol..

[95] Pombo-Suarez M., Sanchez-Piedra C., Gómez-Reino J., Lauper K., Mongin D., Iannone F., Pavelka K., Nordström D.C., Inanc N., Codreanu C. (2023). After JAK inhibitor failure: To cycle or to switch, that is the question–Data from the JAK-pot collaboration of registries. Ann. Rheum. Dis..

[96] Montastruc F., Flumian C., Degboe Y., Constantin A., Ruyssen-Witrand A. (2022). OP0268 Comparison of major cardiovascular and thromboembolic events in safety reports between rheumatoid arthriritis patients treated with JAK inhibitors versus anti-TNF: Results from VigiBase. Ann. Rheum. Dis..

[97] Hoisnard L., Pina Vegas L., Dray-Spira R., Weill A., Zureik M., Sbidian E. (2023). Risk of major adverse cardiovascular and venous thromboembolism events in patients with rheumatoid arthritis exposed to JAK inhibitors versus adalimumab: A nationwide cohort study. Ann. Rheum. Dis..

[98] Gouverneur A., Avouac J., Prati C., Cracowski J.L., Schaeverbeke T., Pariente A., Truchetet M.E. (2022). JAK inhibitors and risk of major cardiovascular events or venous thromboembolism: A self-controlled case series study. Eur. J. Clin. Pharmacol..

[99] Roodenrijs N.M.T., de Hair M.J.H., van der Goes M.C., Jacobs J.W.G., Welsing P.M.J., van der Heijde D., Aletaha D., Dougados M., Hyrich K.L., McInnes I.B. (2018). Characteristics of difficult-to-treat rheumatoid arthritis: Results of an international survey. Ann. Rheum. Dis..

[100] Studenic P., Radner H., Smolen J.S., Aletaha D. (2012). Discrepancies between patients and physicians in their perceptions of rheumatoid arthritis disease activity. Arthritis Rheum..

[101] Ochi S., Sonomoto K., Nakayamada S., Tanaka Y. (2022). Preferable outcome of Janus kinase inhibitors for a group of difficult-to-treat rheumatoid arthritis patients: From the FIRST Registry. Arthritis Res. Ther..

[102] Solomon D.H., Reed G.W., Kremer J.M., Curtis J.R., Farkouh M.E., Harrold L.R., Hochberg M.C., Tsao P., Greenberg J.D. (2015). Disease activity in rheumatoid arthritis and the risk of cardiovascular events. Arthritis Rheumatol..

[103] Molander V., Bower H., Frisell T., Askling J. (2021). Risk of venous thromboembolism in rheumatoid arthritis, and its association with disease activity: A nationwide cohort study from Sweden. Ann. Rheum. Dis..

[104] Baecklund E., Iliadou A., Askling J., Ekbom A., Backlin C., Granath F., Catrina A.I., Rosenquist R., Feltelius N., Sundström C. (2006). Association of chronic inflammation, not its treatment, with increased lymphoma risk in rheumatoid arthritis. Arthritis Rheum..

[105] Mehta B., Pedro S., Ozen G., Kalil A., Wolfe F., Mikuls T., Michaud K. (2019). Serious infection risk in rheumatoid arthritis compared with non-inflammatory rheumatic and musculoskeletal diseases: A US national cohort study. RMD Open.

[106] Gómez-Reino J.J., Carmona L., BIOBADASER Group (2006). Switching TNF antagonists in patients with chronic arthritis: An observational study of 488 patients over a four-year period. Arthritis Res. Ther..

[107] Smolen J.S., Burmester G.R., Combe B., Curtis J.R., Hall S., Haraoui B., van Vollenhoven R., Cioffi C., Ecoffet C., Gervitz L. (2016). Head-to-head comparison of certolizumab pegol versus adalimumab in rheumatoid arthritis: 2-year efficacy and safety results from the randomised EXXELERATE study. Lancet.

[108] Emery P., Keystone E., Tony H.P., Cantagrel A., van Vollenhoven R., Sanchez A., Alecock E., Lee J., Kremer J. (2008). IL-6 receptor inhibition with tocilizumab improves treatment outcomes in patients with rheumatoid arthritis refractory to anti-tumour necrosis factor biologicals: Results from a 24-week multicentre randomised placebo-controlled trial. Ann. Rheum. Dis..

[109] Genovese M.C., Becker J.C., Schiff M., Luggen M., Sherrer Y., Kremer J., Birbara C., Box J., Natarajan K., Nuamah I. (2005). Abatacept for rheumatoid arthritis refractory to tumor necrosis factor alpha inhibition. N. Engl. J. Med..

[110] Gottenberg J.E., Brocq O., Perdriger A., Lassoued S., Berthelot J.M., Wendling D., Euller-Ziegler L., Soubrier M., Richez C., Fautrel B. (2016). Non-TNF-targeted biologic vs a second anti-TNF drug to treat rheumatoid arthritis in patients with insufficient response to a first anti-TNF drug: A randomized clinical trial. JAMA.

[111] Migliore A., Pompilio G., Integlia D., Zhuo J., Alemao E. (2021). Cycling of tumor necrosis factor inhibitors versus switching to different mechanism of action therapy in rheumatoid arthritis patients with inadequate response to tumor necrosis factor inhibitors: A Bayesian network meta-analysis. Ther. Adv. Musculoskelet. Dis..

[112] Tanaka Y., Fautrel B., Keystone E.C., Ortmann R.A., Xie L., Zhu B., Issa M., Patel H., Gaich C.L., de Bono S. (2019). Clinical outcomes in patients switched from adalimumab to baricitinib due to non-response and/or study design: Phase III data in patients with rheumatoid arthritis. Ann. Rheum. Dis..

[113] Raj R., Thomas S., Gorantla V. (2022). Accelerated atherosclerosis in rheumatoid arthritis: A systematic review. F1000Research.

[114] Ketfi C., Boutigny A., Mohamedi N., Bouajil S., Magnan B., Amah G., Dillinger J.G. (2021). Risk of venous thromboembolism in rheumatoid arthritis. Jt. Bone Spine.

[115] Wilton K.M., Matteson E.L. (2017). Malignancy incidence, management, and prevention in patients with rheumatoid arthritis. Rheumatol. Ther..

[116] Listing J., Gerhold K., Zink A. (2013). The risk of infections associated with rheumatoid arthritis, with its comorbidity and treatment. Rheumatology.

[117] Taylor P.C., Takeuchi T., Burmester G.R., Durez P., Smolen J.S., Deberdt W., Issa M., Terres J.R., Bello N., Winthrop K.L. (2022). Safety of baricitinib for the treatment of rheumatoid arthritis over a median of 4.6 and up to 9.3 years of treatment: Final results from long-term extension study and integrated database. Ann. Rheum. Dis..

[118] Bieber T., Feist E., Irvine A.D., Harigai M., Haladyj E., Ball S., Deberdt W., Issa M., Grond S., Taylor P.C. (2022). A review of safety outcomes from clinical trials of baricitinib in rheumatology, dermatology and COVID-19. Adv. Ther..

[119] Thomas K., Vassilopoulos D. (2020). Infections in patients with rheumatoid arthritis in the era of targeted synthetic therapies. Mediterr. J. Rheumatol..

[120] Yamanaka H., Askling J., Berglind N., Franzen S., Frisell T., Garwood C., Greenberg J.D., Ho M., Holmqvist M., Novelli Horne L. (2017). Infection rates in patients from five rheumatoid arthritis (RA) registries: Contextualising an RA clinical trial programme. RMD Open.

[121] Curran D., Callegaro A., Fahrbach K., Neupane B., Vroling H., van Oorschot D., Yawn B.P. (2022). Meta-regression of herpes zoster incidence worldwide. Infect. Dis. Ther..

[122] Kay J., Harigai M., Rancourt J., Dickson C., Melby T., Issa M., de la Torre I., Isaka Y., Cardoso A., Saifan C. (2020). Changes in selected haematological parameters associated with JAK1/JAK2 inhibition observed in patients with rheumatoid arthritis treated with baricitinib. RMD Open.

[123] Taylor P.C., Weinblatt M.E., Burmester G.R., Rooney T.P., Witt S., Walls C.D., Issa M., Salinas C.A., Saifan C., Zhang X. (2019). Cardiovascular safety during treatment with baricitinib in rheumatoid arthritis. Arthritis Rheumatol..

[124] Yates M., Mootoo A., Adas M., Bechman K., Rampes S., Patel V., Qureshi S., Cope A.P., Norton S., Galloway J.B. (2021). Venous thromboembolism risk with JAK inhibitors: A meta-analysis. Arthritis Rheumatol..

[125] Maqsood M.H., Weber B.N., Haberman R.H., Lo Sicco K.I., Bangalore S., Garshick M.S. (2022). Cardiovascular and venous thromboembolic risk with Janus kinase inhibitors in immune-mediated inflammatory diseases: A systematic review and meta-analysis of randomized trials. ACR Open Rheumatol..

[126] Taylor P.C., Bieber T., Alten R., Witte T., Galloway J., Deberdt W., Issa M., Haladyj E., De La Torre I., Grond S. (2023). Baricitinib safety for events of special interest in populations at risk: Analysis from randomised trial data across rheumatologic and dermatologic indications. Adv. Ther..

[127] European Medicines Agency (2023). Janus Kinase Inhibitors (JAKi). https://www.ema.europa.eu/en/medicines/human/referrals/janus-kinase-inhibitors-jaki.

[128] US Food and Drug Administration (2021). FDA Requires Warnings about Increased Risk of Serious Heart-Related Events, Cancer, Blood Clots, and Death for JAK Inhibitors that Treat Certain Chronic Inflammatory Conditions. https://www.fda.gov/drugs/drug-safetyand-availability/fda-requires-warnings-about-increasedrisk-serious-heart-related-events-cancer-blood-clotsand-death.

[129] Charles-Schoeman C., Buch M.H., Dougados M., Bhatt D.L., Giles J.T., Ytterberg S.R., Koch G.G., Vranic I., Wu J., Wang C. (2022). Risk of major adverse cardiovascular events with tofacitinib versus tumour necrosis factor inhibitors in patients with rheumatoid arthritis with or without a history of atherosclerotic cardiovascular disease: A post hoc analysis from ORAL Surveillance. Ann. Rheum. Dis..

[130] Karpouzas G., Szekanecz Z., Baecklund E., Mikuls T., Bhatt D., Shi H., Wang C., Sawyerr G., Chen Y., Menon S. (2022). Relationship between disease activity and adverse events of interest in patients with RA on tofacitinib or TNF inhibitors: Post hoc analysis of a phase 3b/4 randomized safety study. Arthritis Rheumatol..

[131] Frisell T., Baecklund E., Bengtsson K., Di Giuseppe D., Forsblad-d’Elia H., Askling J., on behalf of the ARTIS Study Group (2018). Patient characteristics influence the choice of biological drug in RA, and will make non-TNFi biologics appear more harmful than TNFi biologics. Ann. Rheum. Dis..

[132] Kremer J.M., Bingham C.O., Cappelli L.C., Greenberg J.D., Madsen A.M., Geier J., Rivas J.L., Onofrei A.M., Barr C.J., Pappas D.A. (2021). Postapproval comparative safety study of tofacitinib and biological disease-modifying antirheumatic drugs: 5-year results from a United States-based rheumatoid arthritis registry. ACR Open Rheumatol..

[133] Khosrow-Khavar F., Kim S.C., Lee H., Lee S.B., Desai R.J. (2022). Tofacitinib and risk of cardiovascular outcomes: Results from the Safety of TofAcitinib in Routine care patients with Rheumatoid Arthritis (STAR-RA) study. Ann. Rheum. Dis..

[134] Gouverneur A., Avouac J., Prati C., Cracowski J.L., Schaeverbeke T., Pariente A., Truchetet M.E. (2022). JAK inhibitors and risk of cancer. Arthritis Rheumatol..

[135] Khosrow-Khavar F., Desai R.J., Lee H., Lee S.B., Kim S.C. (2022). Tofacitinib and risk of malignancy: Results from the Safety of Tofacitinib in Routine Care patients with Rheumatoid Arthritis (STAR-RA) study. Arthritis Rheumatol..

[136] Choi S.R., Shin A., Ha Y.J., Lee Y.J., Lee E.B., Kang E.H. (2022). Risk of infections between JAK inhibitors and TNF inhibitors among patients with rheumatoid arthritis. Arthritis Rheumatol..

[137] Heutz J., de Jong P.H.P. (2021). Possibilities for personalised medicine in rheumatoid arthritis: Hype or hope. RMD Open.

[138] Rutherford A.I., Patarata E., Subesinghe S., Hyrich K.L., Galloway J.B. (2018). Opportunistic infections in rheumatoid arthritis patients exposed to biologic therapy: Results from the British Society for Rheumatology Biologics Register for Rheumatoid Arthritis. Rheumatology.

[139] European Medicines Agency Summary of Product Characteristics, Humira, INN-Adalimumab. https://www.ema.europa.eu/en/documents/product-information/humira-epar-product-information_en.pdf.

[140] European Medicines Agency Summary of Product Characteristics, Enbrel, INN-Etanercept. https://www.ema.europa.eu/en/documents/product-information/enbrel-epar-product-information_en.pdf.

[141] European Medicines Agency Summary of Product Characteristics, Remicade, INN-Infliximab. https://www.ema.europa.eu/en/documents/product-information/remicade-epar-product-information_en.pdf.

[142] European Medicines Agency Summary of Product Characteristics, Cimzia, INN-Certolizumab Pegol. https://www.ema.europa.eu/en/documents/product-information/cimzia-epar-product-information_en.pdf.

[143] European Medicine Agency SIMPONI (Golimumab). https://www.ema.europa.eu/en/medicines/human/EPAR/simponi.

[144] Tardella M., Di Carlo M., Carotti M., Ceccarelli L., Giovagnoni A., Salaffi F. (2022). A retrospective study of the efficacy of JAK inhibitors or abatacept on rheumatoid arthritis-interstitial lung disease. Inflammopharmacology.

[145] Kilcher G., Hummel N., Didden E.M., Egger M., Reichenbach S., GetReal Work Package 4 (2018). Rheumatoid arthritis patients treated in trial and real world settings: Comparison of randomized trials with registries. Rheumatology.

[146] Voshaar M.O., Ten Klooster P., Tedjo D., Van de Laar C., Van de Laar M. (2023). Baricitinib versus TNF-inhibitors in patients with active rheumatoid arthritis and an inadequate response to csDMARDs: 12 weeks results of a pragmatic, multicenter, open label, noninferiority trial. POS0830. Ann. Rheum. Dis..

[147] Van De Laar C.J., Oude Voshaar M.A.H., Fakhouri W.K.H., Zaremba-Pechmann L., De Leonardis F., De La Torre I., Van De Laar M.A.F.J. (2020). Cost-effectiveness of a JAK1/JAK2 inhibitor vs a biologic disease-modifying antirheumatic drug (bDMARD) in a treat-to-target strategy for rheumatoid arthritis. ClinicoEconomics Outcomes Res..

