# Supplementary material for: A JAK Inhibitor for Treatment of Rheumatoid Arthritis: The Baricitinib Experience
Source: J Clin Med. 2023 Jul 6;12(13):4527. doi: 10.3390/jcm12134527 (PMC10342289; doi:10.3390/jcm12134527)
Supplement: Supplementary file 1 [file jcm-12-04527-s001.zip › jcm-2464835-supplementary.pdf]

Supplementary Materials

Title: A JAK Inhibitor for Treatment of Rheumatoid Arthritis: The Baricitinib Experience

Author List: Peter C. Taylor[1], Cedric Laedermann[2], Rieke Alten[3], Eugen Feist[4], Ernest Choy[5], Ewa Haladyj[2], Inmaculada De La Torre[2], Pascal Richette[6], Axel Finckh[7] and Yoshiya Tanaka[8]

Affiliations:

[1] Botnar Research Centre, Nuffield Department of Orthopaedics, Rheumatology, and Musculoskeletal Sciences, University of Oxford, Oxford OX3 7LD, UK

[2] Eli Lilly and Company, Indianapolis 46285, Indiana, USA

[3] Internal Medicine II, Rheumatology, SCHLOSSPARK-KLINIK, University Medicine Berlin, Berlin 14059 , Germany

[4] Department of Rheumatology, Helios Clinic Vogelsang-Gommern, cooperation partner of the Otto-von-Guericke University Magdeburg, Magdeburg 39245 , Germany

[5] Division of Infection and Immunity, Cardiff University School of Medicine, Cardiff CF14 4YS, UK

[6] Hôpital Lariboisière, Service de Rhumatologie, Paris, France and Université de Paris, Inserm, UMR-S 1132, Bioscar, Paris, France

[7] Division of Rheumatology, Department of Medicine, Geneva University Hospitals, Geneva 1205 , Switzerland

[8] First Department of Internal Medicine, University of Occupational and Environmental Health, Kitakyushu 807-0804, Japan

Corresponding author:

Peter C. Taylor

Botnar Research Centre,

Nuffield Department of Orthopaedics, Rheumatology, and Musculoskeletal Sciences,

University of Oxford,

Windmill Road,

Oxford, OX3 7LD

United Kingdom

Supplementary Table S1. Baseline demographics and characteristics of patients baricitinib-treated patients in real-world settings and RCTs.

	Patients, n[a]	Age, yrs (mean)	Disease duration, yrs (mean)	Disease severity (mean)	Bio-naïve (%)	3rd line or more (%)	Dosage, 2 mg/day (%)	Comorbidities (%)
**RWE**								
**Baricitinib registry/multicentre**								
**ORBIT-RA** (Spain) [60]	182	62.2	14.6		22.0	36.8	9.9	2.4 CCI
**RABBIT** (Germany) [67]	117/286[b]	59/59[b]	11/14[b]	4.7/4.[4b] DAS28-ESR	38/5[b]	25/58[b,c]	11/17[b]	NR
**BIO-REG** (Austria) [69]	74	64	14.1	3.8 DAS28-ESR	18.9	37.8	NR	65% overall<br>(25% of which was heart disease)
**BSRBR-RA** (UK) [31]	561	60.0	13.1	5.7 DAS28-ESR	46.0	NR	15	NR
**IPS** (Italy) [64]	446	59	9	4.7 DAS28-CRP;<br>25.8 CDAI	34.0	NR		12.5 cardiomyopathy;<br>29.1 hypercholesterolaemia;<br>8.5 diabetes
**ANSWER** (Japan) [70]	166	60.2	12.6	4.3 DAS28-ESR	22.3	54.2	NR	NR
**FIRST** (Japan) [71]	138	57.2	6.4[d]	5.3 DAS28-ESR	32.6	24.6	11.6	NR
**Iwamoto et al.** (Japan) [75]	81	66[d]	11[d]	5.1 DAS28-ESR[d];<br>18 CDAI[d]	22.2	NR	NR	NR
**BIO-1** (Spain) [61]	63	63.0	7.5	NR	NR	NR	NR	NR
**SCQM-RA** (Switzerland) [62,63]	273	59	13	NR	NR	NR	NR	NR
**ARTIS** (Sweden) [65]	1,420	61[d]	13[d]	4.7 DAS28-ESR[d];<br>20 CDAI[d]	NR	NR	NR	29.8 mood disorders;<br>9.9 diabetes; 7.9 cancer;<br>5.5 severe infection;<br>4.7 obstructive lung disease; 3.0 VTE
**ARTIS** (Sweden) [79]	1,837	61	13.2	4.3 DAS28	15	NR	NR	8.0 diabetes; 4.4 cancer;<br>15.1 severe infection;

								4.2 obstructive lung disease
**ARTIS** (Sweden) [80]	1,825	NR	NR	NR	NR	NR	NR	NR
**DANBIO** (Denmark) [66]	275	58.8	13.7	4.4 DAS28	3.3	NR	NR	NR
**TBCR-RA** (Japan) [73,74]	113	66.1	14.0	3.5 DAS28-CRP	28.9	NR	NR	NR
**Baricitinib single centre**								
**LTHT** (England) [81]	69	55.8	14[d]	NR	14.5	NR	NR	NR
**ERLANGEN** (Germany) [82]	139	58.4	9.7	4.3 DAS28-ESR; 4.0 DAS28-CRP	39.6	NR	NR	11.9 diabetes; 40.7 hypertension
	93 (monotherapy)	59.5	9.6	4.3 DAS28-ESR; 4.0 DAS28-CRP	41.9	NR	NR	14.6 diabetes; 44.9 hypertension
**Careggi University Hospital, Florence** (Italy) [83]	43	56.1	12.6	5.3 DAS28-ESR; 4.7 DAS28-CRP	27.9		4 mg/day (100)	NR
**AMIENS** (France) [84]	55	58.0[d]	11.0[d]	4.2 DAS28-ESR[d]; 4.2 DAS28-CRP[d]	NR	NR	NR	2.0 CCI
**Southampton study** (UK) [85]	83	55.3	14.1	NR	NR	NR	NR	NR
**Daegu** (Korea) [86]	20	53.5[d]	7.1[d]	5.6 DAS28-ESR[d]	75.0	NR	NR	5.0 diabetes; 15.0 hypertension
**Sapienza University of Rome** (Italy) [78]	59	58.1[d]	12.0[d]	4.7 DAS28-CRP[d]; 24 CDAI[d]	15.3	64.4	0	NR
**Baricitinib administrative claims data**								
**IT-NHS 2019** (Italy) [88]	445	59.2	8.2	NR	63.6	17.1	NR	0.1 CCI
**IT-NHS 2018** (Italy) [87]	149	57.6	NR	NR	51.0	8.1	NR	NR
**JAKi registry**								
**BIOBADASER 3.0** (Spain) [89]	NR	NR	NR	NR	NR	NR	NR	NR
**BIOBADASAR 3.0** (Argentina) [90]	4,817	NR	NR	NR	NR	NR	NR	NR
**SCQM** (Switzerland) [91]	81	NR	15[d]	3.7 DAS28-CRP;	27.2	39.5	NR	NR

				16.8 CDAI				
**ARTIS** (Sweden) [80]	2150	60[d]	13[d]	4.7 DAS28-ESR[d];<br>4.4 DAS28-CRP[d]	NR	NR	NR	10 diabetes
**OPAL** (Australia) [92]	3,850	NR	NR	NR	NR	NR	NR	NR
**RABBIT** (Germany) [68]	2,030	59.9	12.6	4.2 DAS28-ESR	NR	NR	NR	2.9 sum of comorbidities
**RABBIT** (Germany) [93]	NR	>70[e]	NR	NR	NR	NR	NR	NR
**FIRST** (Japan) [72]	387	58.1	7.3[d]	26.0 CDAI	NR	NR	NR	NR
**JAKi pooled registries**								
**JAK-Pot** (Multinational) [94]	9,329	57.6	13.7	4.7 DAS28;<br>23.7 CDAI	28.2	48.9	NR	52.7 with ≥1 comorbidity
**JAK-Pot** (Multinational) [95]	2,000	NR	NR	NR	NR	NR	NR	NR
**JAKi administrative claims data**								
**VIGIBASE** (Multinational) [96]	39,097	60.6	NR	NR	NR	NR	NR	NR
**SNDS** (France) [97]	8,481	59.3	NR	NR	33.0	22.9	NR	10.8 diabetes; 22.3 hypertension; 4.3 malignancy
	Baricitinib: 5065	NR	NR	NR	NR	NR	20.5	NR
**SNDS** (France) [98]	5,870 (baricitinib: 3,110; RA: 5,497)	58.0	NR	NR	NR	NR	NR	NR
**Baricitinib observational study**								
**PMS study** (Japan) [76]	4,731	63.9			28.9		35	
**RA-BE-REAL** (Multinational) six months [77]	509	59.1	9.1	5.2 DAS28	48.1			
**B023** (Multinational: multi-source meta-analysis) [34]	7,606	NR	NR	NR	NR	NR	NR	NR
**Clinical trials**								
**RA-BEAM** (International) [6]	487	54	10	6.5 DAS28-ESR;	100	0	0	NR

				5.8 DAS28-CRP				
**RA-BEACON** (International) [8]	351	56.0	14.0	6.6 DAS28-ESR;<br>5.9 DAS28-CRP	0	NR	50	NR

Data are reported as mean values or percentages, unless otherwise indicated.

[a]Number of patients receiving baricitinib or JAKi; patients could be included in multiple studies.

[b]Patients treated with baricitinib and new to RABBIT/Switchers to baricitinib while in RABBIT.

[c]Fourth-line or more.

[d]Median values.

[e]Analysis only included patients aged >70 years.

ANSWER, Kansai Consortium for Well-being of Rheumatic Disease Patients (major university-related hospitals: Kyoto University, Osaka University, Osaka Medical College, Kansai Medical University, Kobe University, Nara Medical University and Osaka Red Cross Hospital); ARTIS, Anti-Rheumatic Therapy in Sweden (the Swedish Rheumatology Quality [SRQ] register); BIO-REG: Australian Registry for Biologicals, Biosimilars and tsDMARDs in Rheumatology; B023, metanalysis of patients coming from registries (CorEvitas US, ARTIS, CorEvitas Japan) and administrative claim (Aetna/Healthagen, Anthem [HIRD], HealthVerity PS20, Humana, Marketscan, MDR, Optium Clinformatics, PharMetrics Plus, BKK, JMDC and SNDS); BKK, Betriebskrankenkasse; BSRBR-RA, British Society for Rheumatology Biologics Register for Rheumatoid Arthritis; CCI, Charlson Comorbidity Index; CDAI, Clinical Disease Activity Index; DAS28, Disease Activity Score in 28 joints; DAS28-CRP, DAS28-C-reactive protein; DAS28-ESR, DAS28-erythrocyte sedimentation rate; HIRD, HealthCore Integrated Research Database; IPS, Italian prospective study in 11 centres (Division of Rheumatology and Clinical Immunology, Humanitas Clinical and Research Center – IRCCS, Rozzano, Milan; Rheumatology, Azienda Ospedaliera Universitaria Integrata, Verona, University of Verona; Rheumatology, IRCCS San Raffaele Scientific Institute, Milan and Vita-Salute San Raffaele University, Milan; Rheumatology, Azienda Sanitaria Universitaria Integrata, Udine and University of Udine; Internal Medicine, AOU San Giovanni di Dio e Ruggi d'Aragona, Salerno; Rheumatology, Policlinico Le Scotte, University of Siena; Rheumatology, Allergy and Clinical Immunology, Department of Medicina dei Sistemi, University of Rome Tor Vergata; Rheumatology and Clinical Immunology, Spedali Civili, Brescia; Rheumatology, ASST Papa Giovanni XXIII, Bergamo; Rheumatology, San Gerardo Hospital, Monza; Humanitas University, Department of Biomedical Sciences, Pieve Emanuele, Milan; Rheumatology, ASL3 Genovese, Genova, Italy); IT-NHS 2018, administrative databases from selected Italian settings, including the 'beneficiaries' database' containing patients' demographic data and 'pharmaceutical databases' (inpatient and out- patient) providing data on prescriptions; IT-NHS 2019, integrated administrative databases from a pool of Italian entities from Veneto, Marche, Abruzzo, Apulia and Calabria Regions; JMDC, JMDC, Inc.'s claims database; LTHT, Leeds Teaching Hospitals NHS Trust database; MDR, military health system data repository; OPAL, Optimising Patient outcome in Australian Rheumatology; PMS, post-marketing surveillance – mandated in Japan; RCTs, randomised controlled trials; RWE, real-world evidence; SCQM, Swiss Clinical Quality Management in Rheumatic Diseases Foundation; SNDS, Système National des Données de Santé (French national health data system); TBCR, Tsurumai Biologics Communication Registry; VTE, venous thromboembolism.
